# Dynamic *O*-GlcNAcylation coordinates ferritinophagy and mitophagy to activate ferroptosis

**DOI:** 10.1038/s41421-022-00390-6

**Published:** 2022-05-03

**Authors:** Fan Yu, Qianping Zhang, Hanyu Liu, Jinming Liu, Song Yang, Xiaofan Luo, Wei Liu, Hao Zheng, Qiqi Liu, Yunxi Cui, Guo Chen, Yanjun Li, Xinglu Huang, Xiyun Yan, Jun Zhou, Quan Chen

**Affiliations:** 1 The State Key Laboratory of Medicinal Chemical Biology and Frontier of Science Center for Cell Response, College of Life Sciences, Nankai University, Tianjin, China; 2 Institute of Biophysics, Chinese Academy of Sciences, Beijing, China

**Keywords:** Cell death, Glycosylation

## Abstract

Ferroptosis is a regulated iron-dependent cell death characterized by the accumulation of lipid peroxidation. A myriad of facets linking amino acid, lipid, redox, and iron metabolisms were found to drive or to suppress the execution of ferroptosis. However, how the cells decipher the diverse pro-ferroptotic stress to activate ferroptosis remains elusive. Here, we report that protein *O*-GlcNAcylation, the primary nutrient sensor of glucose flux, orchestrates both ferritinophagy and mitophagy for ferroptosis. Following the treatment of ferroptosis stimuli such as RSL3, a commonly used ferroptosis inducer, there exists a biphasic change of protein *O*-GlcNAcylation to modulate ferroptosis. Pharmacological or genetic inhibition of *O*-GlcNAcylation promoted ferritinophagy, resulting in the accumulation of labile iron towards mitochondria. Inhibition of *O*-GlcNAcylation resulted in mitochondria fragmentation and enhanced mitophagy, providing an additional source of labile iron and rendering the cell more sensitive to ferroptosis. Mechanistically, we found that de-*O*-GlcNAcylation of the ferritin heavy chain at S179 promoted its interaction with NCOA4, the ferritinophagy receptor, thereby accumulating labile iron for ferroptosis. Our findings reveal a previously uncharacterized link of dynamic *O*-GlcNAcylation with iron metabolism and decision-making for ferroptosis, thus offering potential therapeutic intervention for fighting disease.

## Introduction

Ferroptosis is a regulated form of necrotic cell death with the characteristic of iron-dependent lipid peroxidation leading to membrane permeabilization^1^. Elegant early studies have shown that small molecule compounds such as Erastin, which inhibits the import of cysteine, or RSL3, which directly targets and inactivates phospholipid peroxidase glutathione peroxidase 4 (GPX4), lead to a collapse of cellular redox homeostasis, lipid peroxidation, and subsequent ferroptosis^1–4^. These studies have linked glutathione metabolism to the defense of lipid peroxidation, and inactivation of such defense systems causes ferroptosis. Recently, numerous other biological processes, including amino acid and polyunsaturated fatty acid metabolism, phospholipids, NADPH, and coenzyme Q10 were found to suppress or to drive ferroptosis^5^. It appears that ferroptosis lies at the intersection of amino acid metabolism, lipid metabolism, and iron metabolism. Emerging evidence has suggested that ferroptosis contributes to a number of pathophysiological conditions, including neurodegenerative diseases, cardiovascular diseases, and cancers^6,7^. These studies have raised the possibility of exploiting and defending against the ferroptotic vulnerability in different pathophysiological conditions.

Redox-active iron is involved in the initiation and amplification of free radical-mediated lipid peroxidation reactions in ferroptosis^8,9^. The landmark study by the Stockwell laboratory has shown that chelation of intracellular labile ferrous iron using deferoxamine (DFO) prevents its reaction with hydrogen peroxide to generate highly toxic hydroxyl radicals (Fenton reaction), thus inhibiting cells from ferroptosis^1,2^. Also, redox-active iron in the form of heme or iron-sulfur clusters can serve as cofactors for numerous enzymes that participate in lipid peroxidation and oxidative phosphorylation^10^. Therefore, iron absorption, storage, efflux, and regulation are tightly coordinated in order to maintain iron homeostasis, ensuring a sufficient iron supply while limiting iron toxicity^11–13^. Iron in the blood binds to transferrin, which is recognized by cell surface transferrin receptors (TfRs), and subsequently internalized via receptor-mediated endocytosis^14^. Internalized iron is then delivered to acidic endosomes and released^15^. The labile iron can be sequestered and stored by ferritin protein complexes, which are ubiquitously expressed heteropolymers composed of heavy chains and light chains. Ferritin can be delivered to lysosomes by binding to its specific cargo receptor NCOA4^16^, and degraded to release the stored iron (referred to as ferritinophagy), which contributes to ferroptosis^17,18^. Knockout of FTH causes the accumulation of labile iron and accelerates ferroptosis^19–21^, while knocking out NCOA4 blocks iron availability and prevents ferroptosis^19^. These studies highlight the pivotal role of iron metabolism and ferritinophagy for ferroptosis. However, the precise activation mechanism of the ferritinophagy-dependent ferroptosis pathway in response to diverse stresses remains to be elucidated.

*O*-GlcNAcylation is an important post-translational modification that regulates fundamental cellular processes ranging from gene transcription and translation to protein localization, interaction, and degradation^22,23^. Unlike other glycosylation, *O*-GlcNAcylation involves the attachment of a single *O*-linked *N*-acetylglucosamine (*O*-GlcNAc) moiety to Ser or Thr residues of cytoplasmic, nuclear, and mitochondrial proteins. Only a single pair of enzymes regulate this reversible modification: *O*-GlcNAc transferase (OGT), which adds a GlcNAc moiety to proteins; and *O*-GlcNAcase (OGA), which removes the modification from proteins^23^. One of the core functions of *O*-GlcNAcylation is that it can serve as a nutrient sensor of glucose flux through the hexosamine biosynthetic pathway (HBP)^24^. Virtually, all metabolic pathways influence the cellular concentrations of UDP-GlcNAc, the donor of *O*-GlcNAcylation, and *O*-GlcNAcylation serves as a “rheostat” to tune the pathways and processes to accommodate nutrient status and cellular stress^22,25^. Indeed, *O*-GlcNAcylation is highly dynamic and often occurs transiently in response to almost all types of environmental and physiological stresses^22^. *O*-GlcNAcylation has apparently paradoxical roles in diabetes and neurodegeneration. In acute situations, increased *O*-GlcNAcylation is protective, preventing tissue damage in infarcted heart tissue^26^. However, when *O*-GlcNAcylation is chronically elevated, as occurs in diabetes, the sugar modification contributes directly to cardiomyopathy^26^. Indeed, disruption of *O*-GlcNAcylation homeostasis has been implicated in the pathogenesis of many diseases including diabetes, tumor, and neurodegeneration^25,27,28^. Whether and how *O*-GlcNAcylation also responds to pro-ferroptotic stress remains to be explored. Here we demonstrate that *O*-GlcNAcylation senses the ferroptotic stress and coordinates both ferritinophagy and mitophagy for ferroptosis. Our results uncover a previously uncharacterized link of metabolic stresses with cellular iron homeostasis and mitochondria contributing to ferroptosis.

## Results

### *O*-GlcNAcylation plays a critical role in ferroptosis

**Fig. 1:**
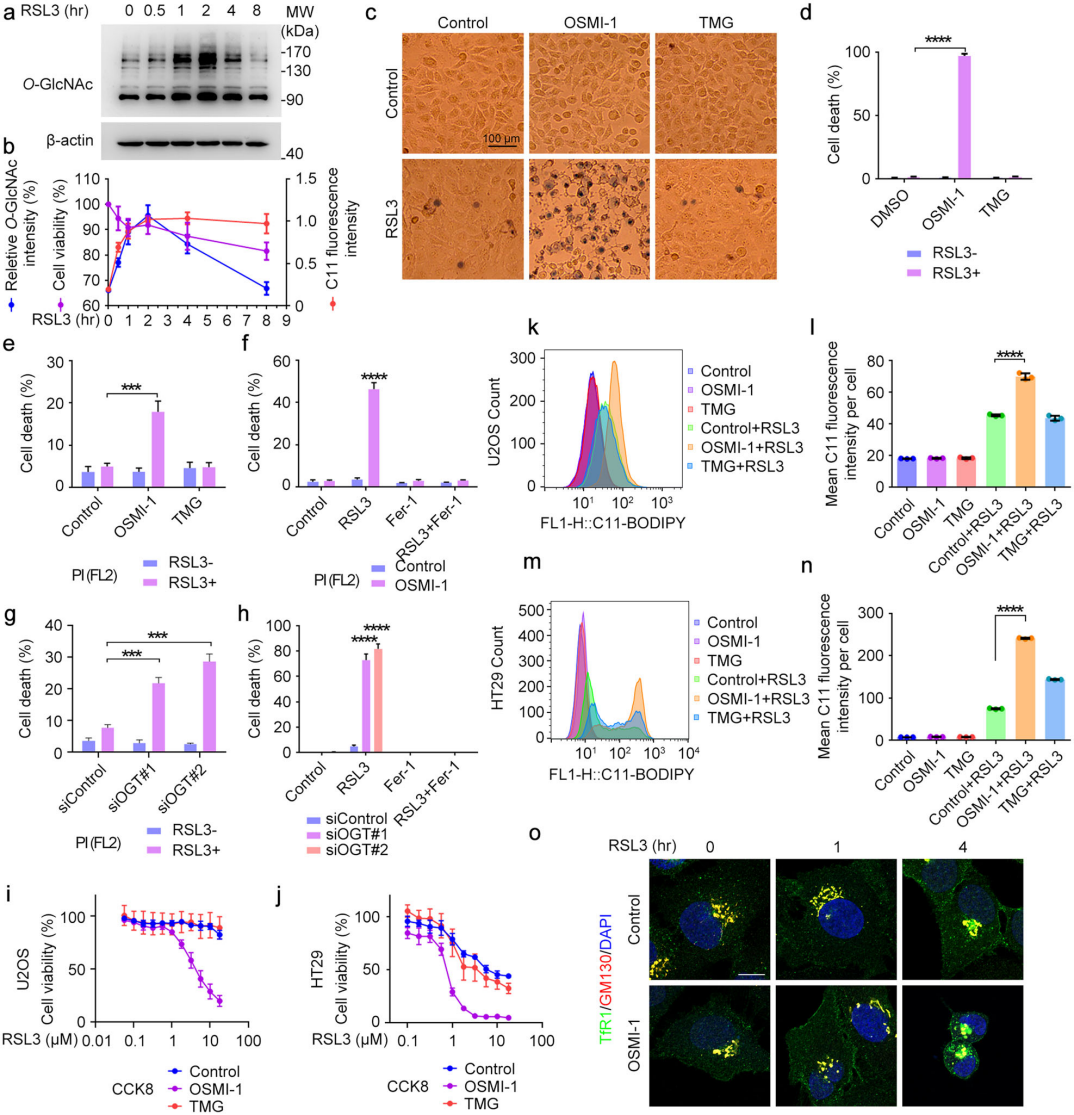
*O*-GlcNAcylation regulates the sensitivity of cells to ferroptosis. **a**, **b** U2OS cells were treated with ferroptosis inducer RSL3 (10 μM) for the indicated time. *O*-GlcNAcylation levels were detected by immunoblotting with antibodies against *O*-GlcNAc and β-actin (**a**), and the relative intensity (*O*-GlcNAc/β-actin) was quantified (**b**). Cell viability was measured by CCK8 (**b**). Lipid ROS production was assessed by BODIPY 581/591 C11 staining followed by flow cytometry (**b**). **c**, **d** U2OS cells were treated with OSMI-1 (30 μM) or TMG (10 μM) for 12 h, and then induced with or without RSL3 for 6 h. Cells were stained with Trypan blue solution (**c**) and the percentages of dead cells were quantified (**d**). **e** U2OS cells were treated with OSMI-1 or TMG for 12 h, and then induced with or without RSL3 for 3 h. Cells were stained with PI and assessed by flow cytometry. **f** U2OS cells were incubated with DMSO or OSMI-1 for 12 h and then pre-treated with Fer-1 for 2 h before the treatment of RSL3 for 4 h. Cells were stained with PI and cell death was then assessed by flow cytometry. **g** U2OS cells were transfected with control or OGT siRNAs for 48 h, and then treated with RSL3 for 3 h before being assessed by PI staining by flow cytometry. **h** U2OS cells were transfected with control or OGT siRNAs for 48 h and then pre-treated with Fer-1 for 2 h before the treatment of RSL3 for 6 h. Cells were stained with Trypan blue and the percentages of dead cells were quantified. **i**, **j** U2OS (**i**) or HT29 cells (**j**) were incubated with OSMI-1 or TMG for 12 h followed by co-treatment of increasing concentrations of RSL3 for 3 h. Dose-dependent cell viability was measured by CCK8 (OD 450). **k**–**n** U2OS or HT29 cells were pre-incubated with OSMI-1 or TMG for 12 h followed by co-treated with RSL3 (10 μM for U2OS,1 μM for HT29) for 2 h. Lipid ROS production was assessed by BODIPY 581/591 C11 staining followed by flow cytometry (Ex/Em: 488/510 nm for the oxidized dye). **o** U2OS cells were treated with DMSO (Control) or OSMI-1 for 12 h, and then treated with RSL3 for different time as indicated. Cells were then subjected to immunofluorescence microscopy with antibodies against TfR1 and GM130. All experiments were repeated at least three times. Scale bars, 10 μm unless specifically indicated. ***P < 0.001, ****P < 0.0001. Error bars indicate SD.

Like phosphorylation, protein *O*-GlcNAcylation at serine and threonine residues responds to almost every type of cellular stress^24,29–31^. We wondered if *O*-GlcNAcylation may be involved in regulating ferroptosis. To address this intriguing question, we first examined the protein *O*-GlcNAcylation during ferroptosis. Immunoblotting analysis revealed a biphasic *O*-GlcNAcylation pattern following treatment with RSL3, a commonly used ferroptosis inducer^4^. The level of *O*-GlcNAcylation increased robustly and reached the peak at 2 h after RSL3 treatment, then declined gradually during ferroptosis. The increase of *O*-GlcNAcylation was accompanied with the accumulation of peroxidized lipids which also reached the peak at 2 h (Fig. 1a, b). This finding was further confirmed by other ferroptosis inducers including ML210^32^ (another GPX4-specific inhibitor, Supplementary Fig. S1a, b), iFSP1(Supplementary Fig. S1c, d), and Erastin (Supplementary Fig. S1e, f). Genetic inhibition of GPX4 by RNAi also induced the accumulation of protein *O*-GlcNAcylation after 48 h followed by a decline at 72 h (Supplementary Fig. S1g, h). Similar patterns were also found in different cell lines such as HUVEC and HT1080 (Supplementary Fig. S1i–l). Interestingly, the increase of *O*-GlcNAcylation caused by RSL3 was hindered by Liproxstatin-1, indicating that the response of *O*-GlcNAcylation was partly due to lipid peroxidation accumulation (Supplementary Fig. S1m, n). In accordance with the increase of *O*-GlcNAcylation, the level of UDP-GlcNAc decreased at the early time point of RSL3 treatment (Supplementary Fig. S1o, p). At the same time, the mRNA and protein level of GFPT1, the most important rate-limiting enzyme of HBP responsible for UDP-GlcNAc synthesis, increased to compensate for the decrease of UDP-GlcNAc (Supplementary Fig. S1q, r).

We reasoned that this initial increase of *O*-GlcNAcylation level might be a strategy employed by the cells to resist ferroptosis. It is thus reasonable to conjecture that inhibiting *O*-GlcNAcylation may promote ferroptosis. To test this assumption, we treated the cell with OSMI-1, a widely used specific inhibitor of OGT, to decrease the *O*-GlcNAcylation level. While OSMI-1 alone has no effect on ferroptosis, it dramatically enhanced the sensitivity to ferroptosis induced by RSL3. U2OS cells were relatively insensitive to ferroptosis and RSL3 alone could only induce <10% cell death (10 μM RSL3, 6 h). However, pretreatment with OSMI-1 increased the percentage of cell death to almost 100% (Fig. 1c, d). We also used an OGA inhibitor, TMG, as a comparison. Increasing the *O*-GlcNAcylation level by TMG showed no significant effect on ferroptosis in U2OS cells (Fig. 1c, d), likely due to the relatively high basal level of *O*-GlcNAcylation, which might be sufficient to confer ferroptosis resistance. Notably, it was recently reported that increasing the *O*-GlcNAcylation level also enhanced the sensitivity to ferroptosis via the YAP pathway in liver cancer^33^. In fact, we also found a similar effect with long-term TMG treatment or OGA knockdown in U2OS cells (data not shown). It was common that OGT and OGA inhibition/knockout showed similar phenotypes because of the disruption of *O*-GlcNAcylation cycle, and feedback effect between OGT and OGA. Similarly, OSMI-1 could also increase sensitivity to other ferroptosis inducers including the system x_c_^−^ inhibitors erastin and sorafenib (Supplementary Fig. S1s, t). Cell death determined by Propidium Iodide staining followed by flow cytometry could also manifest the powerful impact of OSMI-1 to ferroptosis (Fig. 1e). The cell death enhanced by OSMI-1 was strongly inhibited by ferrostatin-1 (Fer-1) (Fig. 1f). Besides the pharmacological inhibition of OGT, we also used siRNA specifically targeted to *OGT* to knock down its expression (Supplementary Fig. S1u). As expected, knocking down OGT rendered U2OS cells more sensitive to RSL3 (Fig. 1g) which can be blocked by Fer-1 (Fig. 1h; Supplementary Fig. S1v). Furthermore, OSMI-1 pretreatment enhances RSL3-induced cytotoxicity in U2OS and HT29 cells in a dose-dependent fashion (Fig. 1i, j).

As lipid peroxidation is a characteristic of ferroptosis^5^, we wondered whether the levels of lipid peroxidation could be also influenced by inhibition of *O*-GlcNAcylation. We used the lipid peroxidation-sensitive dye BODIPY 581/591 C11 (C11-BODIPY) to detect peroxidized lipids within cells by flow cytometry and found that OSMI-1 alone failed to alter lipid peroxidation; however, it remarkably enhances RSL3-induced lipid peroxidation in both U2OS and HT29 cells (Fig. 1k–n). This result could be explained by the existence of a repair system in cells such as GPX4, the pharmacological target of RSL3, which could remove the peroxidized lipids under physiological conditions. Furthermore, TfR1 translocation from Golgi to the plasma membrane was used as a hallmark of ferroptosis^34^. OSMI-1 treatment strongly promoted RSL3-induced translocation of TfR1 at multiple time points (Fig. 1o). Taken together, our results demonstrate that there was biphasic regulation of cellular *O*-GlcNAcylation, and that decreased *O*-GlcNAcylation can increase the sensitivity to ferroptosis.

### Inhibition of *O*-GlcNAcylation promotes ferritinophagy

**Fig. 2:**
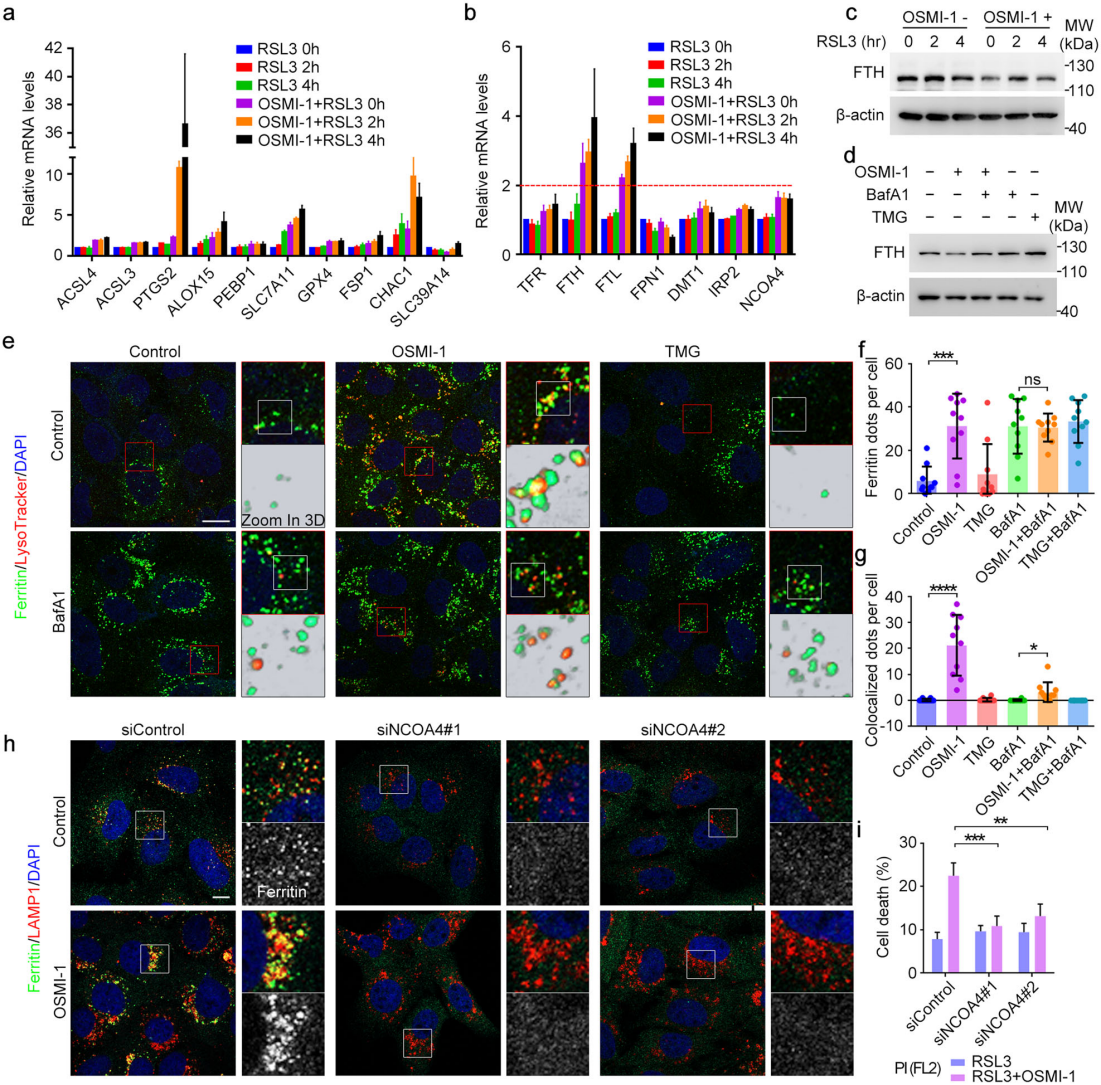
Inhibition of *O*-GlcNAcylation promotes ferritinophagy. **a**–**c** U2OS cells were treated with DMSO (Control) or OSMI-1 for 12 h, and then treated with RSL3 for different time as indicated. The relative mRNA levels were assessed by q-RT-PCR (**a**, **b**). Cells were lysed and immunoblotted with antibodies against FTH and β-actin (**c**). **d** U2OS cells were treated as indicated for 12 h and cell lysates were subjected to immunoblotting with antibodies against FTH and β-actin. **e**–**g** U2OS cells were treated as indicated for 24 h, stained with LysoTracker and then subjected to immunofluorescence microscopy with antibodies against Ferritin (**e**). Ferritin dots per cell (**f**) and co-localized dots of ferritin and LysoTracker per cell (**g**) were quantified. **h** U2OS cells were transfected with control or NCOA4 siRNAs for 48 h and subjected to immunofluorescence microscopy with antibodies against Ferritin and LAMP1. **i** U2OS cells were transfected with control or NCOA4 siRNAs for 48 h and treated with DMSO or OSMI-1 for 12 h, and then induced with RSL3 for 3 h. Cells were stained with PI and assessed by flow cytometry. Scale bars, 10 μm. *P < 0.05, **P < 0.01, ***P < 0.001, ****P < 0.0001; ns, not significant. Error bars indicate SD.

We next sought to understand the underlying mechanism of how inhibition of *O*-GlcNAcylation promotes ferroptosis. We first examined the changes to the ferroptosis-related gene profile induced by RSL3 together with OSMI-1. Real-time PCR analysis showed that the mRNA levels of ferroptosis indicators including *PTGS2* (encoding cyclooxygenase-2, COX-2)^4^ and ChaC glutathione specific gamma-glutamylcyclotransferase 1 (*CHAC1*)^35^ were increased dramatically upon OSMI-1 and RSL3 co-treatment (Fig. 2a). Interestingly, we found that the mRNA levels of ferritin heavy chain (FTH) and light chain (FTL) were both significantly upregulated upon OSMI-1 treatment alone (purple column) or simultaneous treatment with RSL3 (orange and black columns) (Fig. 2b), suggesting their critical roles in this progress.

We then examined the protein level of FTH and found that, compared to the control, OSMI-1 treatment reduced the amount of FTH (Fig. 2c; Supplementary Fig. S2a, b). It is known that FTH and FTL form a heteropolymer to cage iron, and we noticed that the FTH could not be lysed to a monomer but only to a hexamer, even with the strongest lysis buffer (including detergents such as Triton, Sodium Deoxycholate and SDS). Thus, we took the most obvious band at 110–130 kD to represent FTH levels in this manuscript (Supplementary Fig. S2a). Given the fact that mRNA levels of FTH were increased and protein levels were decreased, we assumed that FTH might be degraded via ferritinophagy upon OSMI-1 and RSL3 treatment as previously reported^16^. To confirm this assumption, we utilized BafA1, an autophagy inhibitor, to inhibit ferritinophagy and found that BafA1 could inhibit the decrease of FTH caused by OSMI-1, indicating that decreased *O*-GlcNAcylation promoted ferritinophagy and FTH degradation (Fig. 2d; Supplementary Fig. S2c). This conclusion was further confirmed by knocking down OGT (Supplementary Fig. S2d).

To directly visualize the effect of *O*-GlcNAcylation on ferritinophagy, we co-stained ferritin by a specific antibody with LysoTracker, the indicator of lysosomes. OSMI-1 treatment alone dramatically increased the aggregations of ferritin and the colocalization with lysosomes, while TMG had no such effect. Notably, although BafA1 treatment alone also increased ferritin aggregation to the same level, most of these ferritin aggregation dots were not co-localized with lysosomes (Fig. 2e–g). The localization of overexpressed GFP-FTH to lysosomes was also increased by OSMI-1 treatment (Supplementary Fig. S2e). This result was confirmed in other cell lines including HeLa and HUVEC (Supplementary Fig. S2f, g). NCOA4 is the cargo receptor of ferritin-mediated ferritinophagy, and depletion of NCOA4 could almost completely block ferritin colocalization to lysosomes (Fig. 2h, upper row; Supplementary Fig. S2h). The increase of ferritin localization to lysosomes induced by OSMI-1 was also abolished by knocking down NCOA4 (Fig. 2h, lower row). This suggests that OSMI-1 promoted ferritinophagy by accelerating NCOA4-dependent transport of ferritin to lysosomes. In addition, the increased sensitivity to ferroptosis induced by OSMI-1 could be partially blocked by NCOA4 depletion (Fig. 2i), indicating that the promotion of NCOA4-dependent ferritinophagy contributed to ferroptosis. Together, these results suggest that decreased *O*-GlcNAcylation promotes ferritinophagy and thus increases sensitivity to ferroptosis of cells.

### Inhibition of *O*-GlcNAcylation enhances the mobilization of cellular ferrous iron

**Fig. 3:**
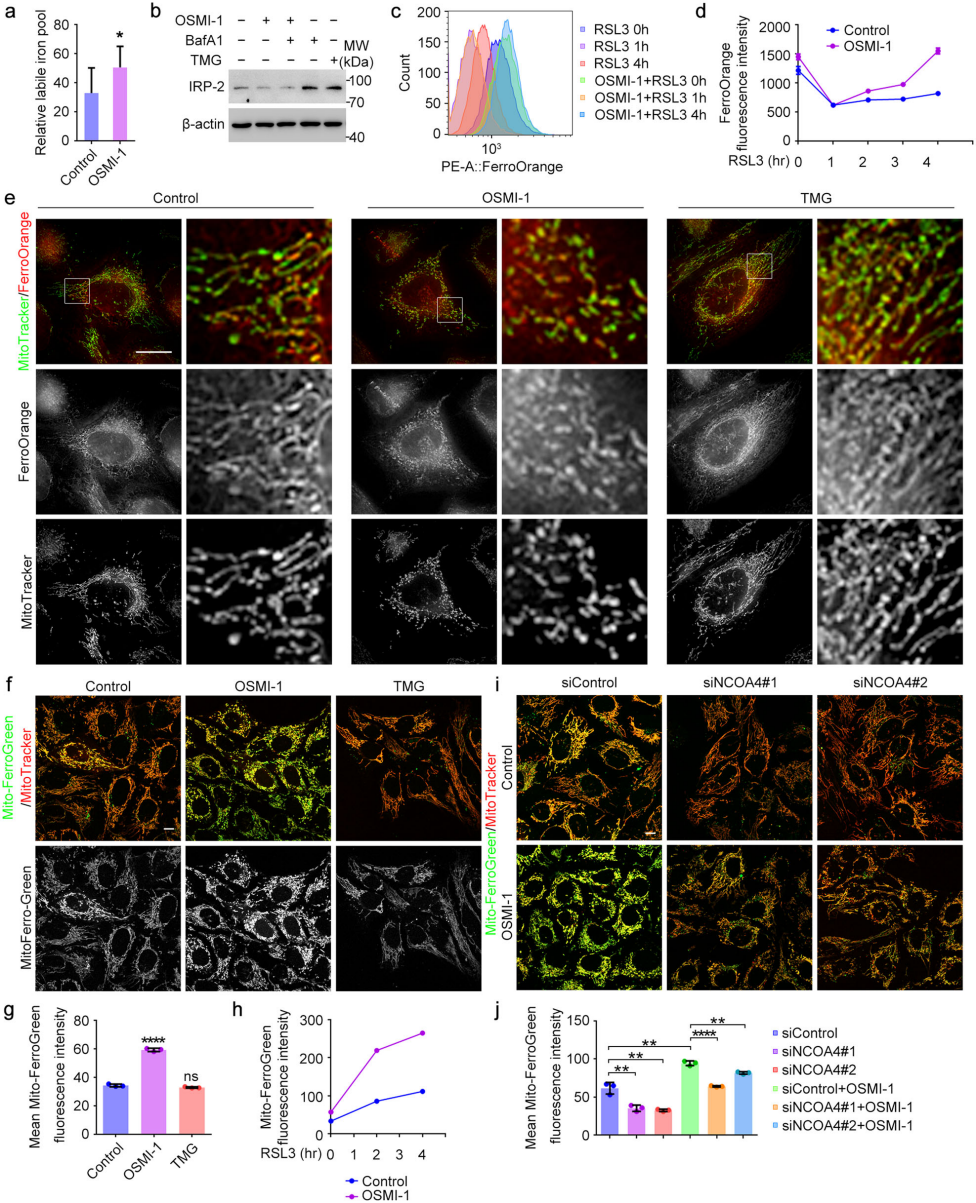
*O*-GlcNAcylation regulates cellular iron flux. **a** U2OS cells were treated with DMSO (Control) or OSMI-1 for 24 h, and the relative cellular labile iron levels were assessed using Calcein-AM. **b** U2OS cells were treated as indicated for 24 h and cell lysates were subjected to immunoblotting with antibodies against IRP2 and β-actin. **c**, **d** U2OS cells were treated with DMSO (Control) or OSMI-1 for 12 h, and then treated with RSL3 for different time as indicated. The levels of cellular ferrous iron were assessed by flow cytometry using FerroOrange (**c**). Then the mean FerroOrange fluorescence intensity of each cell was quantified (**d**). **e** U2OS cells were treated with OSMI-1 or TMG for 24 h, and the cellular ferrous iron levels were assessed by 3D**-**structured illumination microscopy using FerroOrange. **f** U2OS cells were treated with OSMI-1 or TMG for 24 h, and the mitochondrial ferrous iron levels were assessed by confocal microscopy using Mito-FerroGreen. **g** U2OS cells were treated with OSMI-1 or TMG for 24 h, and the mitochondrial ferrous iron levels were assessed by flow cytometry using Mito-FerroGreen. Then the mean Mito-FerroGreen fluorescence intensity of each cell was quantified. **h** U2OS cells were treated with DMSO (Control) or OSMI-1 for 12 h, and then induced with RSL3 for different time as indicated. The mitochondrial ferrous iron levels were assessed by flow cytometry using Mito-FerroGreen. **i** U2OS cells were transfected with control or NCOA4 siRNAs for 48 h, and treated with DMSO (Control) or OSMI-1 for 24 h. The mitochondrial ferrous iron levels were assessed by confocal microscopy using Mito-FerroGreen. **j** U2OS cells were treated as described in **i**. The mitochondrial ferrous iron levels were assessed by flow cytometry using Mito-FerroGreen and then quantified. Scale bars, 10 μm. *P < 0.05, **P < 0.01, ****P < 0.0001. Error bars indicate SD.

Next, we investigated whether the OSMI-1-induced promotion of ferritinophagy led to more free iron release, accounting for the enhanced lipid peroxidation and ferroptosis. We used Calcein-AM to detect the relative level of labile iron^36^, and found an increase of cellular labile iron levels after OSMI-1 treatment (Fig. 3a). To confirm this finding, we examined the level of iron-responsive-element-binding protein 2 (IRP2), which directly responds to cellular iron levels^37^, and found that OSMI-1 treatment decreased IRP2 abundance to an extent comparable with control, indicating an increase of cellular iron levels after OSMI-1 treatment (Fig. 3b).

Ferroptosis is specifically caused by ferrous iron, so we sought to detect the overall level of cellular ferrous iron by FerroOrange staining^38^ (Supplementary Fig. S3a). Surprisingly, we found a robust decline of ferrous iron content immediately after RSL3 treatment (Fig. 3c, d, 1 h), indicating an initial response of the cells to sequester iron to resist ferroptosis. The ferrous iron levels then rebounded slowly during the onset of detectable ferroptosis (Fig. 3c, d, Control). Interestingly, compared to RSL3 treatment alone, pretreatment with OSMI-1 led to an increased basal level of cellular ferrous iron levels (Fig. 3c, d, 0 h), and following RSL3 treatment, the recovery of the ferrous iron level after the initial decline was much faster as compared with control, suggesting an increased releasing rate of cellular iron with decreased *O*-GlcNAcylation level (Fig. 3c, d, 2–4 h). This result was confirmed by fluorescent imaging (Supplementary Fig. S3b, c).

Strikingly, microscopic analysis revealed that most ferrous iron was localized to mitochondria as indicated by its colocalization with MitoTracker (Fig. 3e; Supplementary Fig. S3a, b). We were thus prompted to investigate the critical role of the mitochondria in this progress. We found a significant increase of mitochondrial ferrous iron indicated by Mito-FerroGreen, a specific dye to detect mitochondrial ferrous iron^39,40^, after OSMI-1 treatment (Fig. 3f, g; Supplementary Fig. S3d). This increase could be further promoted by RSL3 (Fig. 3h; Supplementary Fig. S3e). Interestingly, RSL3 alone also induced a gradual increase of mitochondrial ferrous iron levels during ferroptosis (Fig. 3h; Supplementary Fig. S3e, Control), suggesting that labile iron might be transported to mitochondria to buffer the toxic iron. We also found an increase of mitochondrial ferrous iron levels after OGT knockdown, which was in accordance with OGT inhibition by OSMI-1 (Supplementary Fig. S3f). We assumed that this OSMI-1-induced increase of mitochondrial ferrous iron is resulted from the iron released from promoted ferritinophagy. Indeed, NCOA4 knockdown decreased the mitochondrial ferrous iron levels, and the OSMI-1-induced increase was effectively rescued (Fig. 3i, j; Supplementary Fig. S3g). Taken together, these data suggest decreased *O*-GlcNAcylation enhances the level of cellular labile iron, especially ferrous iron, rendering the cells more sensitive to ferroptosis. In addition, it is possible that mitochondria may serve as a buffering pool for the excessive cellular iron released from ferritinophagy.

### Inhibition of *O*-GlcNAcylation promotes mitochondrial fragmentation and mitophagy

**Fig. 4:**
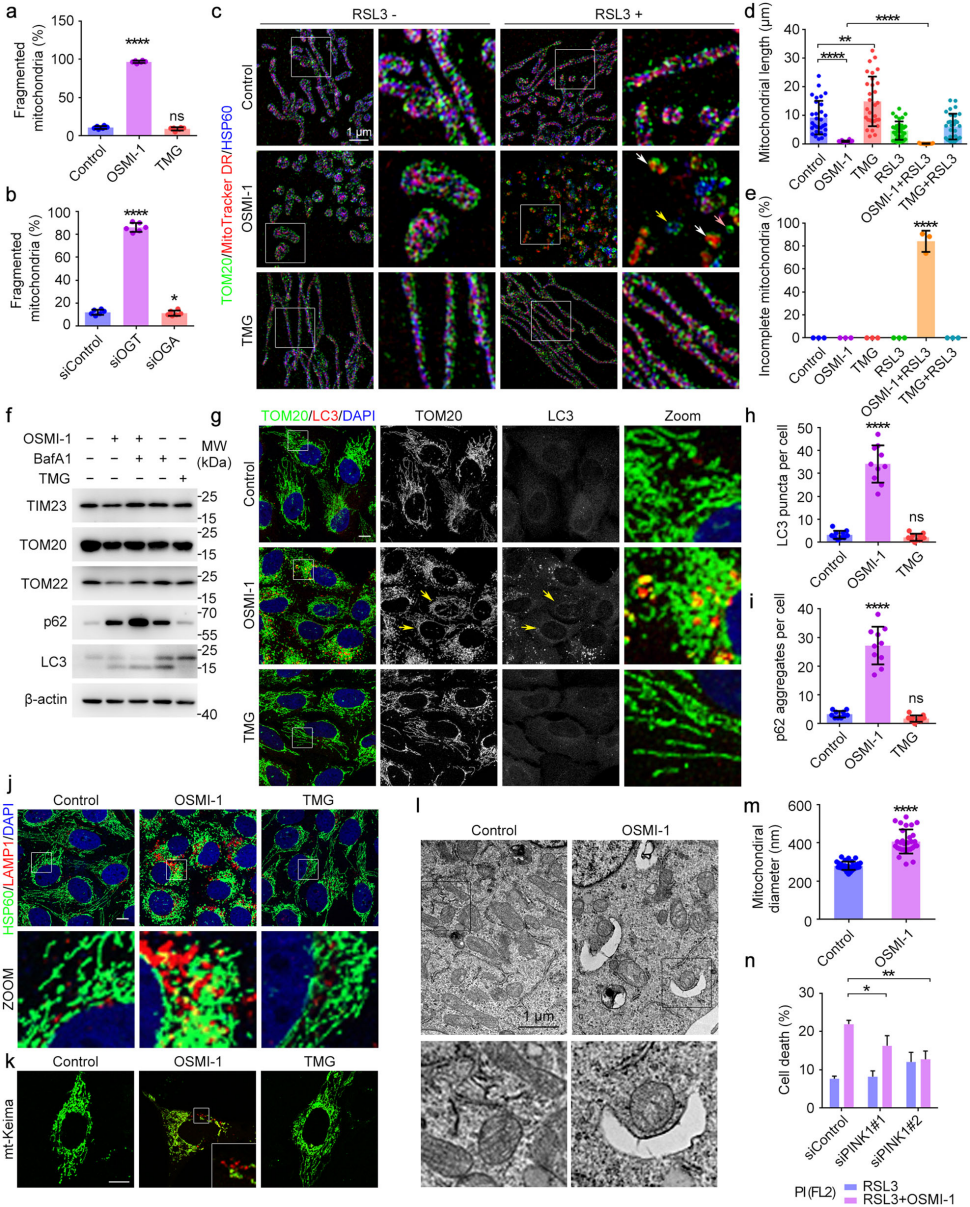
Inhibition of *O*-GlcNAcylation promotes mitophagy. **a**, **b** U2OS cells were treated with OSMI-1 or TMG for 24 h (**a**), or transfected with OGT or OGA siRNAs for 48 hr (**b**). Cells were subjected to immunofluorescence microscopy with antibodies against TOM20, and the percentage of fragmented mitochondria was quantified. **c**–**e** U2OS cells were incubated with OSMI-1 or TMG for 12 h, and treated with or without RSL3 for 6 h. Cells were stained with MitoTracker and subjected to 3D**-**structured illumination microscopy with antibodies against TOM20 (mitochondrial outer membrane) and HSP60 (mitochondrial matrix) (**c**). Mitochondrial length (**d**) and incomplete mitochondria (mitochondria who lost their integrity and lack components such as inner membrane (yellow arrow), matrix (white arrow) or even both (pink arrow)) (**e**) were quantified. **f** U2OS cells were treated as indicated for 24 h and cell lysates were subjected to immunoblotting with indicated antibodies. **g**, **h** U2OS cells were treated with OSMI-1 or TMG for 24 h and subjected to immunofluorescence microscopy with antibodies against TOM20 and LC3 (**g**), and the number of LC3 puncta per cell was quantified (**h**). **i** U2OS cells were treated as described in **h**, and the number of p62 aggregates per cell was quantified. **j** U2OS cells were treated as described in **h**. Cells were stained with antibodies against HSP60 and LAMP1. **k** U2OS cells were transfected with mt-Keima and treated with OSMI-1 or TMG for 24 h, and then assessed with confocal microscopy. **l**, **m** U2OS cells were treated with DMSO or OSMI-1 for 24 h and subjected to TEM (**l**). Mitochondrial diameter was quantified (**m**). **n** U2OS cells were transfected with control or PINK1 siRNAs for 48 h and treated with DMSO or OSMI-1 for 12 h, and then induced with RSL3 for 3 h. Cells were stained with PI and assessed by flow cytometry. *P < 0.05, **P < 0.01, ****P < 0.0001; ns, not significant. Scale bars, 10 μm unless specifically indicated. Error bars indicate SD.

As a large amount of iron released from accelerated ferritinophagy was transported to mitochondria, we tried to figure out how this will impact on mitochondrial behaviors and ferroptosis. We noticed that decreasing *O*-GlcNAcylation level by inhibiting or knocking down OGT led to profound fragmentation of mitochondria (Figs. 3e–i and 4a, b; Supplementary Fig. S4a–d). 3D-Structured Illumination Microscopy (3D-SIM) analysis showed a distinct fragmentation pattern within the cell following OSMI-1 treatment or its combination with RSL3. Although all mitochondria were fragmented after OSMI-1 treatment, the individual mitochondria kept their integrity with an intact outer membrane (indicated by TOM20), inner membrane (indicated by MitoTracker) and matrix (indicated by HSP60). However, during the late phase of ferroptosis, the fragmented mitochondria lost their integrity (Fig. 4c–e). This explains why OSMI-1 treatment alone failed to trigger ferroptosis. As the mitochondrial fragmentation is the prerequisite to mitophagy^41–44^, we reason that these fragmented mitochondria were prone to mitophagy. Indeed, the levels of mitochondrial proteins including TIM23, TOM20, and TOM22 decreased along with decreased *O*-GlcNAcylation levels, which can be inhibited by BafA1. Meanwhile, p62 and type II LC3 were increased, indicating the accelerated mitophagy flux^41^ (Fig. 4f; Supplementary Fig. S4e). This result was further confirmed by the increase of LC3 puncta and p62 aggregates after OSMI-1 treatment (Fig. 4g–i; Supplementary Fig. S4f). Notably, the number of LC3 puncta had a positive correlation with the level of mitochondrial fragmentation (Fig. 4g). These fragmented mitochondria were co-localized with lysosomes, suggesting that they were transported to lysosomes for degradation (Fig. 4j; Supplementary Fig. S4g, h). To specifically monitor mitophagy, we expressed mt-Keima, a pH-sensitive fluorescent protein which presents green fluorescence under neutral environments such as normal mitochondria and presents red fluorescence under acidic pH when mitochondria are engulfed by lysosomes during mitophagy^45^. A far greater number of red dots appeared in the OSMI-1-treated cells transfected with mt-Keima (Fig. 4k), and more autophagosome membrane surrounded mitochondria as revealed by EM analysis (Fig. 4l, m), suggesting an increase of mitophagy. These results indicate that decreased *O*-GlcNAcylation promotes mitophagy, providing an additional source of irons for ferroptosis. Similar results were obtained in other cell lines including HeLa and HUVEC (Supplementary Fig. S4i, j).

To verify that mitophagy might be the underlying mechanism to enhance sensitivity to ferroptosis, we knocked down PINK1^46,47^ and found that blocking mitophagy could partially prevent OSMI-1 and RSL3-induced ferroptosis (Fig. 4n; Supplementary Fig. S4k). Taken together, decreased *O*-GlcNAcylation promotes mitochondrial fragmentation and extensive mitophagy which may release the stored iron, rendering the cells more sensitive to ferroptosis.

### Simultaneous inhibition of ferritinophagy and mitophagy abolishes ferroptosis

**Fig. 5:**
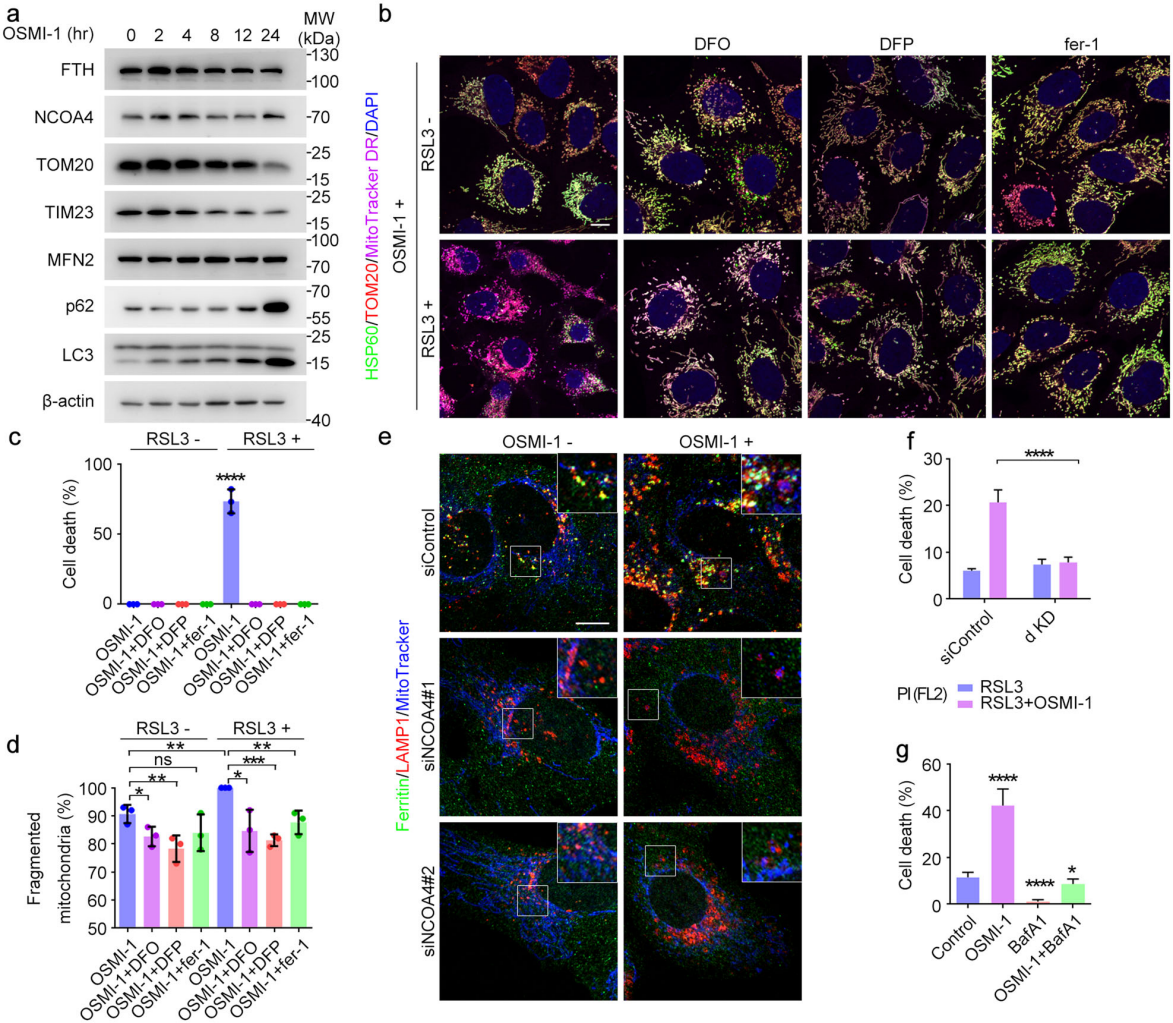
*O*-GlcNAcylation-dependent ferritinophagy and mitophagy contribute to ferroptosis. **a** U2OS cells were treated with OSMI-1 for the indicated time and cell lysates were subjected to immunoblotting with indicated antibodies. **b**–**d** U2OS cells were incubated with OSMI-1 for 12 h, and treated with or without RSL3 for 6 h. Cells were stained with MitoTracker and subjected to immunofluorescence microscopy with antibodies against TOM20 and HSP60 (**b**). The percentage of dead cells (**c**) and fragmented mitochondria (**d**) were quantified. **e** U2OS cells were transfected with control or NCOA4 siRNAs for 48 h and treated with DMSO or OSMI-1 for 12 h. Cells were subjected to immunofluorescence microscopy with antibodies against ferritin and LAMP1. **f** U2OS cells were transfected with control or combination of PINK1 and NCOA4 siRNAs for 48 h and treated with DMSO or OSMI-1 for 12 h, and then induced with RSL3 for 3 h. Cells were stained with PI and assessed by flow cytometry. **g** U2OS cells were treated as indicated for 12 h and then induced with RSL3 for 3 h. Cells were stained with Trypan blue solution and the percentages of dead cells were quantified. Scale bars, 10 μm. *P < 0.05, **P < 0.01, ***P < 0.001, ****P < 0.0001; ns, not significant. Error bars indicate SD.

We have shown that inhibition of *O*-GlcNAcylation promoted both ferritinophagy and mitophagy. We next asked whether there is any interplay between them to promote ferroptosis. Upon OSMI-1 treatment, the degradation of FTH was relatively faster than that of mitochondrial proteins, suggesting that ferritinophagy occurs prior to mitophagy (Fig. 5a; Supplementary Fig. S5a). This result was in accordance with the assumption that some iron released due to ferritinophagy was transported to mitochondria (Fig. 3f–j). We then wondered whether the iron released due to ferritinophagy caused iron overload in mitochondria, resulting in their fragmentation. Iron chelation by DFO or DFP could completely abrogate ferroptosis induced by OSMI-1 and RSL3 without much effect on the OSMI-1-induced fragmentation of mitochondria. This indicates that iron overload may not be the only cause of OSMI-1-induced mitochondrial fragmentation (Fig. 5b–d). NCOA4 knockdown abolished ferritinophagy promoted by OSMI-1 treatment, and it had a limited effect on mitophagy (Fig. 5e), indicating that OSMI-1-induced ferritinophagy and mitophagy were relatively independent.

Given that both ferritinophagy and mitophagy contributed to enhanced sensitivity to ferroptosis, we performed a double knockdown of NCOA4 and PINK1 and found simultaneous inhibition of ferritinophagy and mitophagy almost completely blocked the effect of OSMI-1-enhanced ferroptosis (Fig. 5f; Supplementary Fig. S5b). Inhibition of the general autophagy pathways by BafA1 exhibited similar effects (Fig. 5g). These results further support our notion that *O*-GlcNAcylation regulates both ferritinophagy and mitophagy to dictate ferroptosis.

### De-*O*-GlcNAcylation of FTH at S179 promotes its interaction with NCOA4 and activates ferroptosis

**Fig. 6:**
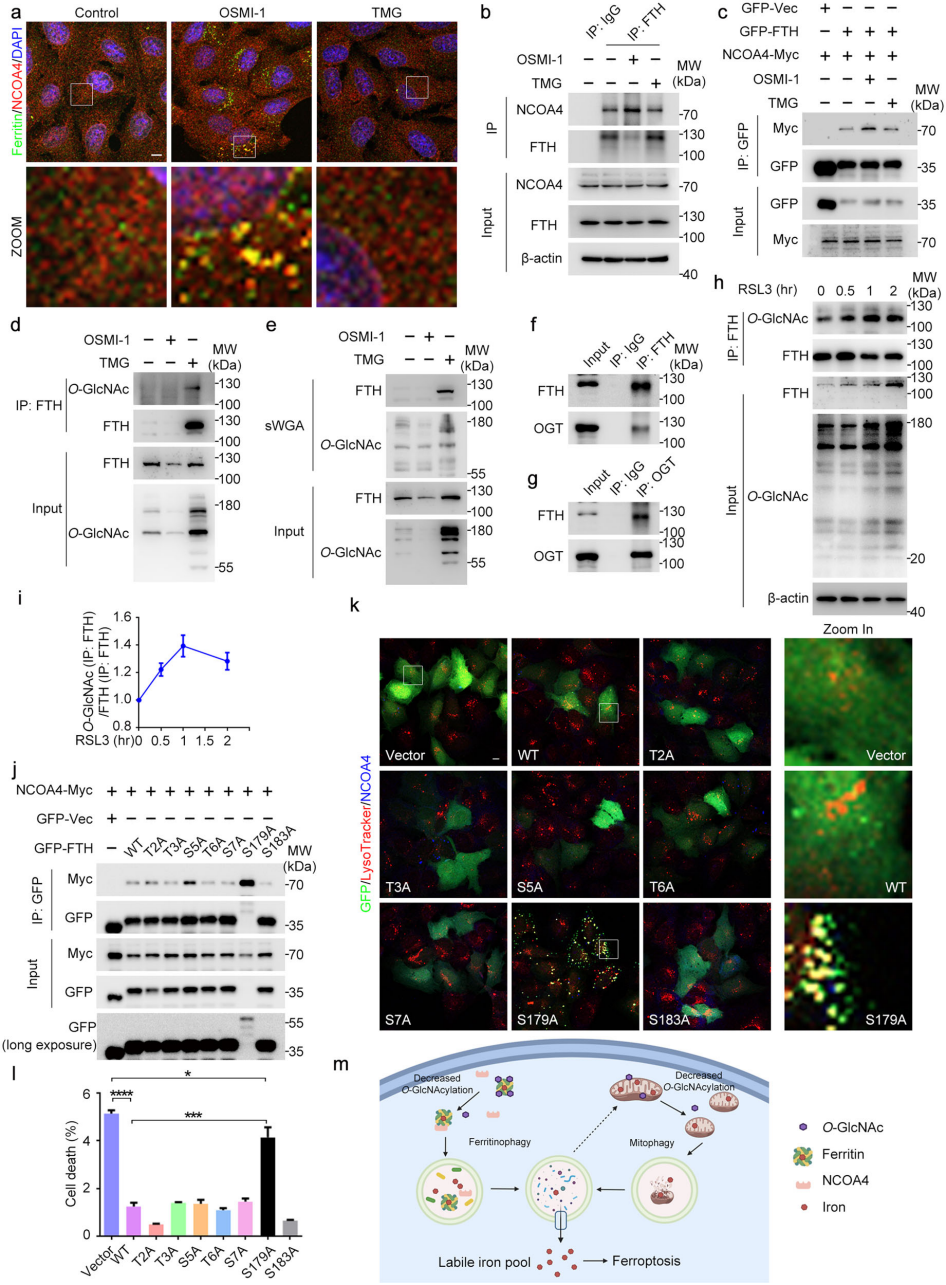
De-*O*-GlcNAcylation of FTH at S179 promotes its interaction with NCOA4. **a** U2OS cells were treated with OSMI-1 or TMG for 24 h and subjected to immunofluorescence microscopy with antibodies against Ferritin and NCOA4. **b** Cell lysates from U2OS cells treated with OSMI-1 or TMG for 24 h were immunoprecipitated with antibodies against NCOA4 or FTH and IgG, and then immunoblotted with the indicated antibodies. **c** Cell lysates from 293T cells stably expressing the indicated proteins were immunoprecipitated with anti-GFP magnetic beads and immunoblotted with the indicated antibodies. **d** Cell lysates from U2OS cells treated with OSMI-1 or TMG for 24 h were immunoprecipitated with antibodies against FTH and immunoblotted with the indicated antibodies. **e** Cell lysates from U2OS cells treated with OSMI-1 or TMG for 24 h were pulled down with sWGA and immunoblotted with the indicated antibodies. **f**, **g** Cell lysates from U2OS cells were immunoprecipitated with antibodies against FTH or OGT and IgG and immunoblotted with the indicated antibodies. **h**, **i** Cell lysates from U2OS cells treated with RSL3 for the indicated time were immunoprecipitated with antibodies against FTH and immunoblotted with the indicated antibodies. The relative optical intensity was quantified. **j** Cell lysates from 293T cells stably expressing the indicated proteins were immunoprecipitated with anti-GFP magnetic beads and immunoblotted with the indicated antibodies. **k** U2OS cells were transfected with indicated plasmids for 24 h. Cells were stained with LysoTracker and immunostained with anti-NCOA4 antibody, and then subjected to confocal microscopy. **l** U2OS cells were transfected with indicated plasmids for 24 h, and incubated with RSL3 for 8 h. Cells were stained with PI and assessed by flow cytometry, and the percentage of GFP/PI double-positive cells was quantified. **m** Model for the regulation of *O*-GlcNAcylation in ferroptosis via ferritinophagy and mitophagy. Inhibition of *O*-GlcNAcylation promoted ferritinophagy by increasing the interaction between FTH and NCOA4, resulting in the accumulation of labile iron which may flux toward mitochondria. Decreased *O*-GlcNAcylation also resulted in mitochondria fragmentation and enhanced mitophagy, providing additional source for labile iron and rendering the cell to be more sensitive to ferroptosis. Scale bars, 10 μm. *P < 0.05, ***P < 0.001, ****P < 0.0001; ns, not significant. Error bars indicate SD.

We next tried to figure out the downstream substrates of *O*-GlcNAcylation. Inhibition of *O*-GlcNAcylation significantly increased the colocalization of ferritin and NCOA4 with lysosomes (Fig. 6a; Supplementary Fig. S6a). We predicted that the interaction between ferritin and NCOA4 was increased by inhibition of *O*-GlcNAcylation. Indeed, immunoprecipitation analysis revealed that their interaction was increased after decreasing *O*-GlcNAcylation by OSMI-1 treatment, OGT knockdown, or OGA overexpression (Fig. 6b, c; Supplementary Fig. S6b, d). To be noticed, increasing *O*-GlcNAcylation by TMG treatment, OGA knockdown, or OGT overexpression had no dramatic effect on the interaction between FTH and NCOA4 (Fig. 6b, c; Supplementary Fig. S6b, c). We assumed that FTH was adequately *O*-GlcNAcylated in the cells. When we increased the *O*-GlcNAcylation level of FTH purified from *E. coli* with no post-translational modification in vitro, the interaction between FTH and NCOA4 was reduced (Supplementary Fig. S6e). Indeed, we confirmed that FTH was *O*-GlcNAcylated in cells (Fig. 6d, e). FTH also showed a strong interaction with OGT (Fig. 6f, g). We assumed that *O*-GlcNAcylation of FTH might respond to ferroptotic stress, so we detected the *O*-GlcNAcylation level of FTH during the early stage of ferroptosis. There was a robust increase in the *O*-GlcNAcylation level of FTH following treatment with RSL3, and this level declined immediately during the onset of the detectable ferroptosis (Fig. 6h, i). To determine the *O*-GlcNAcylation site of FTH, we constructed mutants for every serine and threonine residues of FTH located at both the N- and C-terminus. Strikingly, when S179 was mutated to alanine, the interaction between FTH and NCOA4 was profoundly increased, whereas other mutations had no obvious effect (Fig. 6j). As a result, it seems that the S179A mutant promoted the accumulation and degradation of FTH, because the increased interaction with NCOA4 accelerated the transport to lysosomes. In accordance with this result, the S179A mutant also presented a distinctive localization pattern compared to wild-type FTH and other mutants. All S179A mutant proteins co-localized with NCOA4 and were transported to lysosomes (Fig. 6k). We then checked the *O*-GlcNAcylation level of the wide type and S179A mutant and found a dramatically decrease of *O*-GlcNAcylation when S179 was mutated (Supplementary Fig. S6f, g). Moreover, after S179 was mutated, its interaction with NCOA4 was no longer affected by any change of *O*-GlcNAcylation level (Supplementary Fig. S6h–k), suggesting that S179 was the site regulating their interaction. There was still a slight increase in the *O*-GlcNAcylation level of FTH-S179A following treatment with RSL3 (Supplementary Fig. S6l, m), indicating that there might be other *O*-GlcNAcylation sites on FTH. However, it seems that S179A was the only regulation site of FTH-NCOA4 interaction under this condition. It was possible that *O*-GlcNAcylation of the S179 residue in FTH provided steric hindrance to prevent its interaction with NCOA4, thus limiting the cellular ferritinophagy under physiological conditions. To confirm this possibility, we knocked down NCOA4 and found that the S179A mutant no longer accumulated in lysosomes, indicating that the increase of FTH-S179A accumulation to lysosomes was due to promoted NCOA4-dependent ferritinophagy (Supplementary Fig. S6n). Besides, overexpression of wild-type FTH blocked the RSL3-induced ferroptosis as expected. However, the FTH-S179A mutant, but not other mutants, no longer possessed the protective ability against ferroptosis (Fig. 6l). This suggests that mutation at S179 rendered it to ferritinophagy and/or failed to chelate iron to prevent ferroptosis. Together, these results demonstrate that the *O*-GlcNAcylation of FTH at S179 is of functional importance. De-*O*-GlcNAcylation of FTH at S179 increases its interaction with NCOA4, and thus increases the transport of FTH to lysosomes and promotes ferritinophagy as a result (Fig. 6m).

## Discussion

Great strides have been made over the past few years in the study of iron-dependent ferroptosis. However, the missing link that remains to be addressed is how iron mobilization is regulated in response to ferroptosis stress. In this study, we show that the protein *O*-GlcNAcylation regulates ferritinophagy and mitochondria behaviors to dictate ferroptosis. In response to ferroptosis stimuli such as RSL3, cells may adopt two immediate response strategies to resist ferroptosis. First, ferritin is *O*-GlcNAcylated to block its interactions with NCOA4 to prevent ferritinophagy and labile iron flux into mitochondria (Figs. 3f–g and 6a–c). Second, some cellular labile iron might be transported to mitochondria to buffer the toxic iron. However, persistent pro-ferroptotic stress may inactivate the OGT, leading to de-*O*-GlcNAcylation of ferritin for activation of ferritinophagy which, together with mitophagy, provides a source of labile iron for the subsequent Fenton reaction, the explosive ROS and lipid peroxidation for ferroptosis (Fig. 6m). In support of this, we showed that inhibition of *O*-GlcNAcylation strongly accelerates ferritinophagy, mitochondrial fragmentation, mitophagy, and subsequent ferroptosis. Our findings thus suggest that both *O*-GlcNAcylation-dependent ferritinophagy and mitochondrial alterations are critical decision-making steps from a disease-related iron overload pro-ferroptotic state towards ferroptosis. As the synthesis of UDP-GlcNAc integrates lipid, energy, and nucleotide metabolism^48^, and *O*-GlcNAcylation of proteins rapidly increases when cells are exposed to almost every type of stress, including heat, high salt, heavy metals, UV light, hypoxia, and others^22,49,50^, we argue that *O*-GlcNAcylation represents a key nexus linking mitochondria and iron mobilization with ferroptosis, contributing to related pathophysiological conditions.

De-*O*-GlcNAcylation of FTH for ferritinophagy: *O*-GlcNAcylation serves as a signaling molecule to function as a real-time sensor of glucose and metabolic status by directly modifying enzymatic function, protein–protein interactions or counteracting the phosphorylation^48,51,52^. Such modifications are able to fine-tune processes critical to metazoan physiology including regulating growth, monitoring cellular environmental cues and modulating cell fate^22^. Our findings show for the first time that the de-*O*-GlcNAcylation of FTH at S179 activates ferritinophagy and ferroptosis. Early studies have shown that FTH interacts with NCOA4 for ferritinophagy, which is one of the major sources of labile iron for ferroptosis^17,19^. Knockout of ATG genes blocks the degradation of FTH and prevent ferroptosis^16^. While our findings are consistent with these results, we further revealed that de-*O*-GlcNAcylation provides a new layer of regulation via the interaction of ferritin with NCOA4 and a new mechanism of activation for ferritinophagy and ferroptosis. *O*-GlcNAcylation of FTH under physiological conditions and the increase during the early phase of ferroptosis blocks its interaction with NCOA4 to prevent ferritinophagy and ferroptosis. Conversely, the removal of *O*-GlcNAcylation enhanced the interaction between FTH and NCOA4 for ferritinophagy. As the site for *O*-GlcNAcylation is identical for protein phosphorylation^31^, it is predicted that phosphorylation of FTH may also regulate ferritinophagy and ferroptosis. Further studies are warranted to study this possibility.

Mitochondria as sentinel and iron pool for ferroptosis: previous studies have shown that mitochondria become fragmented and condensed, distinct from other forms of regulated cell death^53^. It is not clear whether such a change is the accompanying events of ferroptosis or mitochondria have a causal role in ferroptosis, nor was the molecular regulation of mitochondrial behaviors in response to ferroptotic stress. Our results suggest a paradoxical role of mitochondria in ferroptosis. During the early stage of ferroptosis, mitochondria may serve as a buffering organelle that sequesters labile iron to prevent ferroptosis. It is suggested that iron from the lysosomes owing to ferritinophagy can be transported into mitochondria via Kiss and Run fashion^54,55^. On the other hand, persistent iron overload may lead to mitochondrial damage, which subsequently (1) activates mitophagy which may provide an additional source of iron for lipid peroxidation^56^; (2) activates the mitochondrial-dependent vicious cycle and directly releases iron, ROS, or even peroxidated lipids to trigger and to amplify ferroptosis^57^. It is interesting to notice that in this study, we found that inhibition of *O*-GlcNAcylation enhanced mitophagy independent of cellular iron contents or ferritinophagy, suggesting that there must be additional mechanisms. A previous study has reported several *O*-GlcNAcylated mitochondrial proteins such as Drp1^58^ and PGK1^59^. It is reasonable to assume that there are other *O*-GlcNAcylated mitochondrial proteins that are directly responsible for mitophagy. Further work is underway to assess this possibility.

It is also interesting to note that mitophagy can be both “friend and foe” for ferroptosis. In response to mild stress or during the early stages of iron overload, mitophagy may sequester iron into mitophagosomes and diminish the source of ROS for ferroptosis. However, extensive mitophagy may provide an additional source of iron to amplify lipid peroxidation and ferroptosis. Thus, inhibition of mitophagy by knockdown of PINK1 prevents, at least in part, de-*O*-GlcNAcylation promoted ferroptosis, and simultaneous inhibition of both pathways almost completely prevents ferroptosis. Apparently, ferritinophagy and mitophagy may crosstalk to regulate iron metabolism, as both are dependent on core autophagy machineries^19,60^, and to provide a source of the iron required for catalyzing lipid peroxidation and ferroptosis.

Implications for diseases related to iron overload: dysregulation of *O*-GlcNAcylation modifications, iron overload, mitochondrial dysfunctions, and ferroptosis are common etiologies of aging and aging-related human diseases, including neurodegenerative diseases^61^, cardiovascular diseases, metabolic syndromes, and cancers^62–64^. Our study suggests that these molecular events are interlinked and thus presents a unified model of how iron metabolism and mitochondria are coordinated in response to diverse stresses for cell fate decisions. Although most measurements were conducted in cultured cells where iron availability can readily be altered, it is reasonable to assume that the pathways also function under pathophysiological conditions. Our findings further suggest that intervention of the pathways described here, including *O*-GlcNAcylation, iron homeostasis, and mitochondrial functions, may be beneficial for the treatments of diseases related to iron overload.

## Materials and methods

### Cell culture

All cell lines were obtained from the American Type Culture Collection and were free of mycoplasma contamination. Cells were cultured in Dulbecco’s modified Eagle’s medium (DMEM) with 10% (volume/volume; v/v) FBS at 37 °C with a humidified atmosphere of 20% O_2_ and 5% CO_2_.

### Antibodies and beads

The following antibodies were used in these studies. *O*-GlcNAc (CTD110.6, Sigma, O7764); OGT (Abcam, ab96718); OGT (sigma, SAB5202310); OGA (Santa Cruz, sc-376429); β-actin (Abcam, ab8226); TfR1(I3-6800, Invitrogen); GFPT1(A3882, ABclonal), FTH (Abcam, ab65080); ferritin (Rockland 200-401-090-0100); NCOA4 (ARA70) (Bethyl Laboratories A302-272A); NCOA4 (Abnova, h00008031-m05); LAMP1 (Developmental Studies Hybridoma Bank, H4A3); IRP2 (Santa Cruz, Sc-33682); TOM20 (BD, 612278); TIM23 (BD, 611223); TOM22 (Proteintech, 11278-1-ap); HSP60 (Cell Signaling, 12165s); MFN2 (Abnova, H00009927-m03); p62 (MBL, m162-3); LC3 (MBL, pm036); PINK1 (Cell Signaling, 6946); GFP (Roche, 11814460001); Myc (Sigma, m4439). Alexa Fluor 488, 568 and 647 secondary antibodies were from Life Technologies, and DAPI was from Sigma-Aldrich.

The following beads were used in these immunoprecipitation analyses: Anti-GFP beads (Abcam, ab193983); Anti-Myc-beads (bimake, B26302); ProteinA/G (Thermo, 20422); sWGA (Vector, AL-1023S).

### Chemicals

OSMI-1 (Sigma, SML1621), Thiamet G (TMG, Sigma, SML0244), and DFP (Sigma, 379409) were from Sigma-Aldrich. RSL3, Erastin, Sorafenib, ferrostatin-1, iFSP1, ML210, and BafA1 were from MCE. Liproxstatin-1 (T2376) was from TargetMol. DFO (B6068) was from APExBio.

### RNAi

siRNAs were transfected using Lipofectamine RNAiMAX (Invitrogen).

The sequences for OGT siRNAs were 5′-GGAUGGAAUUCAUAUCCUU-3′ (#1) and 5′-AUACGAUGGCAUCUUCUGGUAACCC-3′ (#2).

The sequences for OGA siRNAs were 5′-GCAAGAAGAUUGUAUUAGU-3′ (#1) and 5′-GGCACUUUCUGUUAUCCAA-3′ (#2).

The sequences for GPX4 siRNAs were 5′-GTAACGAAGAGATCAAAGA-3′ (#1) and 5′-GAGGCAAGACCGAAGTAAA-3′ (#2).

The sequences for NCOA4 siRNAs were 5′-ACAAAGAUCUAGCCAAUCA-3′ (#1); and 5′-GACCUUAUUUAUCAGCUUA-3′ (#2).

The sequences for PINK1 siRNAs were 5′-CCAACAGGCUCACAGAGAA-3′ (#1); and 5′-GGCUGGUGAUCGCAGCAGAUUU-3′ (#2).

### Plasmids

Mammalian expression plasmids for GFP-FTH, NCOA4-Myc, Flag-DsRed-FTH, GFP-OGT, HA-OGA, and mutants were generated by PCR and site-directed mutagenesis. All constructs were confirmed by DNA sequencing. Plasmids were transfected using Lipo3000 (Invitrogen).

### Immunofluorescence microscopy

Cells grown on glass coverslips were fixed with 4% paraformaldehyde for 30 min and permeabilized in 0.3% Triton X-100/phosphate-buffered saline (PBS) for 1 min. Then cells were blocked in 4% bovine serum albumin (BSA) with 0.1% Triton X-100 and then incubated with primary antibodies, secondary antibodies, and DAPI. Cells were then examined with a Zeiss LSM900 confocal microscope or DeltaVision OMX SR 3D-SIM. The fluorescent intensity was quantified by ImageJ.

### TEM analysis

Cell pellets were fixed in 2.5% glutaraldehyde, post-fixed in 2% osmium tetroxide, dehydrated in gradual ethanol and propylene oxide, embedded in Epon, and cured for 24 h at 60 °C. Ultrathin sections (50 nm) were placed onto 200 mesh copper grids and double-stained with uranyl acetate and lead citrate before transmission electron microscopy analysis (HITACHI HT7700 Exalens).

### Cell death assay

Cells were seeded onto 12-well plates at a density of 50% confluence. The next day, cells were pre-treated with OSMI-1 (30 μM) or TMG (10 μM) for 12 h or transfected with siRNA for 48 h. Subsequently, cells were treated with or without RSL3 for appropriate time. Sometimes, cells were treated with ferrostatin-1 (2 μM) before RSL3. To measure cell death, cells were digested and collected, stained with 5 μg/mL propidium iodide (Sigma) and the percentage of the propidium iodide-positive dead cell population was analyzed using flow cytometer BD FACS Calibur (BD Biosciences) and an FL2 detector. At least 10,000 single cells were analyzed per well and all experiments were carried out at least in triplicate. In some experiments, Trypan blue dye was used to assess cell death coupling with microscopy and cell counting.

### Cell viability assay

Cells were seeded onto 96-well plates at a density of 2 × 10^4^ per well. After pretreatment with OSMI-1 or TMG or transfection with siRNAs, 100 μL fresh medium per well with increasing concentrations of ferroptosis inducers RSL3, Erastin, or Sorafenib in the absence or presence of OSMI-1 or TMG were added to induce ferroptosis. Subsequently, cells were exposed to 10 μL Cell Counting Kit-8 (CCK8, Dojindo) for 1 h at 37 °C, 5% CO_2_ in an incubator. The absorbance at a wavelength of 450 nm was determined using a Multiskan Sky with Touch Screen (Thermo scientific). Cell viability under test conditions is reported as a percentage relative to the negative control treatment. Three wells at least were tested per experiment and all experiments were carried out at least in triplicate.

### Lipid peroxidation measurement

Cells were seeded on 12-well plates and pre-treated with OSMI-1 or TMG for 12 h, followed by co-treatment with RSL3 for 2 h. Then, cells were incubated with 5 μM BODIPY 581/591 C11 (Invitrogen) for 30 min and then washed with PBS twice and harvested by trypsinization and centrifugation. Lipid peroxidation was assessed using the flow cytometer BD FACS Calibur (BD Biosciences) with a 488 nm laser on an FL1 detector for the oxidized probe, because oxidation of the polyunsaturated butadienyl portion of C11-BODIPY resulted in a shift of the fluorescence emission peak from ~590 to ~510 nm proportional to lipid ROS generation. A minimum of 10,000 single cells were analyzed per well. Three independent biological replicates were performed for each condition.

### RT-qPCR

Cells were seeded on 12-well plates for 24 h and cells were pre-treated with or without OSMI-1 for 12 h, followed by co-treatment with 5 μM RSL3 for indicated times. Then, cells were washed twice before being lysed by lysis buffer and the RNAs were extracted using Eastep Super total RNA extraction kit (Promega) according to the manufacturer’s protocol. cDNA was obtained by reverse transcription of 1 μg RNA using High Capacity cDNA Reverse Transcription Kit (Thermo Fisher Scientific). Primers used to amplify are listed in Supplementary Table S1. qPCR reactions were performed using the Power SYBR Green PCR Real Master Mix. Triplicate samples per treatment were analyzed on LightCycler 96 Instrument (Roche). Differences on mRNA levels compared to internal reference ACTB mRNA levels between control and experimental conditions were calculated using DDCt method. Three independent biological replicates were performed for each condition.

### Determination of UDP-GlcNAc by ultrahigh performance LC-MS

Cells were lysed with 0.3 mL 70% (v/v) ice-cold methanol. The extract was vortexed and centrifuged at 16,000 × *g* for 10 min at 4 °C to remove large chunks of debris. The supernatant collected (10 μL injections) were separated using Acquity Ultra Performance LC system. The chromatography was performed using a Waters Acquity UPLC HILIC column (2.1 × 100 mm, 1.7 μm). The mobile phases consisted of H_2_O (A) and acetonitrile (B). The UPLC eluting conditions were optimized as follows: 95% B (0–2 min), 75% B (2–3 min), 55% B (3–6 min), and 95% B (6–8 min). The flow rate was 0.3 mL/min. The column was maintained at 30 °C. The UDP-GlcNAc standard (U4375, Sigma) was used to determine UDP-GlcNAc concentration and composition in cell extracts. Mass spectrometry was performed using Waters Xevo TQ-S operating in negative ion mode.

### *O*-GlcNAcylation assay in vitro

Reaction mixtures containing 5 μg purified human FTH protein (purified from *E. coli* with no *O*-GlcNAcylation), 1 μg purified human OGT (from insect cells) and 5 mM UDP-GlcNAc in a buffer of 50 mM Tris-HCl (pH 7.5), 12.5 mM MgCl_2_ and 1 mM DTT were incubated at 37 °C for 2 h. Then, co-IP was performed with NCOA4-Myc overexpressed and immunoprecipitated from 293T cells.

### Cellular labile iron detection

Cellular labile iron concentrations were measured using Calcein-AM (BD Pharmingen) according to the previously described methods^36,65^. Briefly, cells were seeded onto 96-well plates. After treatment, cells were washed twice with PBS and incubated with 125 nM Calcein-AM for 15 min at 37 °C under dark conditions. The fluorescence intensity at ex495, em515 was detected with a microplate reader. Then 100 μM DFP was added into each well and the fluorescence intensity was detected again. The difference between the fluorescence intensity reflects the relative quantity of cellular free iron levels.

### Cellular and mitochondrial ferrous iron detection

The levels of cellular ferrous iron and mitochondrial ferrous iron were assessed by FerroOrange probes (Dojindo) and Mito-FerroGreen probes (Dojindo) following the instructions provided by Dojindo^39,40^. Briefly, to detect the cellular ferrous iron, cells were plated on coverglass bottom confocal dishes and treated as described. Then cells were washed three times with HBSS, incubated with 1 μM FerroOrange for 30 min at 37 °C, 5% CO_2_ in an incubator, and immediately observed through Zeiss LSM900 confocal microscope or DeltaVision OMX SR microscope. To confirm the specificity of FerroOrange, cells were also co-incubated with FerroOrange and DFP. When detecting the mitochondrial iron, cells were washed three times with HBSS, incubated with 5 μM Mito-FerroGreen and MitoTracker Red for 30 min at 37 °C, 5% CO_2_ in an incubator, and then washed three times with HBSS. Cells were then examined with a Zeiss LSM900 confocal microscope. To detect iron levels by flow cytometry, cells were seeded on 12-well plates, treated as described and collected. Cells were resuspended with FerroOrange and incubated for 30 min, and then assessed using a BD FACS Caliburflow cytometer. For Mito-FerroGreen detection, cells need to be washed an additional three times with HBSS before assessment.

### Immunoblotting and Co-IP

For western blots, cells were lysed using RIPA cell lysis buffer (50 mM Tris-HCl, 1% Triton, 0.1% SDS, 1% sodium deoxycholate, 150 mM NaCl and 1 mM EDTA, pH 7.5) and equal amounts of proteins were separated by SDS-PAGE.

For immunoprecipitation, cells were lysed using IP lysis buffer (20 mM Tris-HCl, 150 mM NaCl, 1 mM EDTA, 1 mM EGTA, 1% NP-40, 2.5 mM Sodium pyrophosphate, 10% glycerol, pH 7.5) and supplemented with protease inhibitor cocktail (Roche). Cell lysates were incubated with beads (primary antibodies were added when using Protein A/G beads) at 4 °C for 4 h, washed five times with IP lysis buffer, and then subjected to immunoblotting.

### Statistical analysis

All quantitative data were presented as the means ± SD of at least three independent experiments by Student’s *t*-test comparing two groups using unpaired two-tailed analysis.

## Supplementary information

supplementary information

Table S1qPCR primers

## References

[1] Dixon SJ (2012). Ferroptosis: an iron-dependent form of nonapoptotic cell death. Cell.

[2] Friedmann Angeli JP (2014). Inactivation of the ferroptosis regulator Gpx4 triggers acute renal failure in mice. Nat. Cell Biol..

[3] Ingold I (2018). Selenium utilization by GPX4 is required to prevent hydroperoxide-induced ferroptosis. Cell.

[4] Yang WS (2014). Regulation of ferroptotic cancer cell death by GPX4. Cell.

[5] Stockwell BR (2017). Ferroptosis: a regulated cell death nexus linking metabolism, redox biology, and disease. Cell.

[6] Conrad M, Angeli JP, Vandenabeele P, Stockwell BR (2016). Regulated necrosis: disease relevance and therapeutic opportunities. Nat. Rev. Drug Discov..

[7] Toyokuni S, Ito F, Yamashita K, Okazaki Y, Akatsuka S (2017). Iron and thiol redox signaling in cancer: an exquisite balance to escape ferroptosis. Free Radic. Biol. Med..

[8] Chen X, Yu C, Kang R, Tang D (2020). Iron metabolism in ferroptosis. Front. Cell Dev. Biol..

[9] Sheftel AD, Zhang AS, Brown C, Shirihai OS, Ponka P (2007). Direct interorganellar transfer of iron from endosome to mitochondrion. Blood.

[10] Richardson, D. R. et al. Mitochondrial iron trafficking and the integration of iron metabolism between the mitochondrion and cytosol. *Proc. Natl. Acad. Sci. USA* **107**, 10775–10782 (2010). 10.1073/pnas.0912925107 PMC2890738 20495089

[11] Ganz T (2008). Iron homeostasis: fitting the puzzle pieces together. Cell Metab..

[12] Crielaard BJ, Lammers T, Rivella S (2017). Targeting iron metabolism in drug discovery and delivery. Nat. Rev. Drug Discov..

[13] Hentze MW, Muckenthaler MU, Galy B, Camaschella C (2010). Two to tango: regulation of mammalian iron metabolism. Cell.

[14] Gao M, Monian P, Quadri N, Ramasamy R, Jiang X (2015). Glutaminolysis and transferrin regulate ferroptosis. Mol. Cell.

[15] Jiang X, Stockwell BR, Conrad M (2021). Ferroptosis: mechanisms, biology and role in disease. Nat. Rev. Mol. Cell Biol..

[16] Mancias JD, Wang X, Gygi SP, Harper JW, Kimmelman AC (2014). Quantitative proteomics identifies NCOA4 as the cargo receptor mediating ferritinophagy. Nature.

[17] Gao M (2016). Ferroptosis is an autophagic cell death process. Cell Res..

[18] Dowdle WE (2014). Selective VPS34 inhibitor blocks autophagy and uncovers a role for NCOA4 in ferritin degradation and iron homeostasis in vivo. Nat. Cell Biol..

[19] Hou W (2016). Autophagy promotes ferroptosis by degradation of ferritin. Autophagy.

[20] Rui T (2021). Deletion of ferritin H in neurons counteracts the protective effect of melatonin against traumatic brain injury-induced ferroptosis. J. Pineal Res..

[21] Fang X (2020). Loss of cardiac ferritin H facilitates cardiomyopathy via Slc7a11-mediated ferroptosis. Circ. Res..

[22] Yang X, Qian K (2017). Protein O-GlcNAcylation: emerging mechanisms and functions. Nat. Rev. Mol. Cell Biol..

[23] Hart GW, Housley MP, Slawson C (2007). Cycling of O-linked beta-N-acetylglucosamine on nucleocytoplasmic proteins. Nature.

[24] Whelan SA, Lane MD, Hart GW (2008). Regulation of the O-linked beta-N-acetylglucosamine transferase by insulin signaling. J. Biol. Chem..

[25] Bond MR, Hanover JA (2013). O-GlcNAc cycling: a link between metabolism and chronic disease. Annu. Rev. Nutr..

[26] Chatham, J. C. & Marchase, R. B. The role of protein O-linked beta-N-acetylglucosamine in mediating cardiac stress responses. *Biochim. Biophys. Acta* **1800**, 57–66 (2010). 10.1016/j.bbagen.2009.07.004 PMC2814923 19607882

[27] Yang X (2008). Phosphoinositide signalling links O-GlcNAc transferase to insulin resistance. Nature.

[28] Slawson C, Hart GW (2011). O-GlcNAc signalling: implications for cancer cell biology. Nat. Rev. Cancer.

[29] Kearse, K. P. & Hart, G. W. Lymphocyte activation induces rapid changes in nuclear and cytoplasmic glycoproteins. *Proc. Natl. Acad. Sci. USA* **88**, 1701–1705 (1991). 10.1073/pnas.88.5.1701 PMC51092 2000378

[30] Zachara NE (2004). Dynamic O-GlcNAc modification of nucleocytoplasmic proteins in response to stress. A survival response of mammalian cells. J. Biol. Chem..

[31] Hart, G. W., Slawson, C., Ramirez-Correa, G. & Lagerlof, O. Cross talk between O-GlcNAcylation and phosphorylation: roles in signaling, transcription, and chronic disease. *Annu. Rev. Biochem.* **80**, 825–858 (2011). 10.1146/annurev-biochem-060608-102511 PMC3294376 21391816

[32] Eaton JK (2020). Selective covalent targeting of GPX4 using masked nitrile-oxide electrophiles. Nat. Chem. Biol..

[33] Zhu G (2021). O-GlcNAcylation enhances sensitivity to RSL3-induced ferroptosis via the YAP/TFRC pathway in liver cancer. Cell Death Discov..

[34] Feng H (2020). Transferrin receptor is a specific ferroptosis marker. Cell Rep..

[35] Magtanong L (2019). Exogenous monounsaturated fatty acids promote a ferroptosis-resistant cell state. Cell Chem. Biol..

[36] Yoshida M (2019). Involvement of cigarette smoke-induced epithelial cell ferroptosis in COPD pathogenesis. Nat. Commun..

[37] Pantopoulos K, Porwal SK, Tartakoff A, Devireddy L (2012). Mechanisms of mammalian iron homeostasis. Biochemistry.

[38] Weber RA (2020). Maintaining iron homeostasis is the key role of lysosomal acidity for cell proliferation. Mol. Cell.

[39] Hirayama T, Kadota S, Niwa M, Nagasawa H (2018). A mitochondria-targeted fluorescent probe for selective detection of mitochondrial labile Fe(ii). Metallomics.

[40] Fujimaki, M. et al. Iron supply via NCOA4-mediated ferritin degradation maintains mitochondrial functions. *Mol. Cell Biol*. **39**, e00010-19 (2019). 10.1128/MCB.00010-19 PMC6597882 31061094

[41] Chakraborty, J. et al. USP14 inhibition corrects an in vivo model of impaired mitophagy. *EMBO Mol. Med.* **10**, e9014 (2018). 10.15252/emmm.201809014 PMC6220287 30249595

[42] Grassi, D. et al. Identification of a highly neurotoxic alpha-synuclein species inducing mitochondrial damage and mitophagy in Parkinson’s disease. *Proc. Natl. Acad. Sci. USA* **115**, E2634–E2643 (2018). 10.1073/pnas.1713849115 PMC5856519 29487216

[43] Sprenger HG, Langer T (2019). The good and the bad of mitochondrial breakups. Trends Cell Biol..

[44] Gomes, L. C. & Scorrano, L. Mitochondrial morphology in mitophagy and macroautophagy. *Biochim. Biophys. Acta* **1833**, 205–212 (2013). 10.1016/j.bbamcr.2012.02.012 22406072

[45] Sun N (2015). Measuring in vivo mitophagy. Mol. Cell.

[46] Vives-Bauza, C. et al. PINK1-dependent recruitment of Parkin to mitochondria in mitophagy. *Proc. Natl. Acad. Sci. USA* **107**, 378–383 (2010). 10.1073/pnas.0911187107 PMC2806779 19966284

[47] Koehler CL, Perkins GA, Ellisman MH, Jones DL (2017). Pink1 and Parkin regulate Drosophila intestinal stem cell proliferation during stress and aging. J. Cell Biol..

[48] Hanover JA, Krause MW, Love DC (2012). Bittersweet memories: linking metabolism to epigenetics through O-GlcNAcylation. Nat. Rev. Mol. Cell Biol..

[49] Matthews, J. A., Acevedo-Duncan, M. & Potter, R. L. Selective decrease of membrane-associated PKC-alpha and PKC-epsilon in response to elevated intracellular O-GlcNAc levels in transformed human glial cells. *Biochim. Biophys. Acta* **1743**, 305–315 (2005). 10.1016/j.bbamcr.2004.11.001 15843043

[50] Kneass ZT, Marchase RB (2004). Neutrophils exhibit rapid agonist-induced increases in protein-associated O-GlcNAc. J. Biol. Chem..

[51] Hanover, J. A., Krause, M. W. & Love, D. C. The hexosamine signaling pathway: O-GlcNAc cycling in feast or famine. *Biochim. Biophys. Acta* **1800**, 80–95 (2010). 10.1016/j.bbagen.2009.07.017 PMC2815088 19647043

[52] Hardiville S, Hart GW (2014). Nutrient regulation of signaling, transcription, and cell physiology by O-GlcNAcylation. Cell Metab..

[53] Nixon, R. A. & Yang, D. S. Autophagy and neuronal cell death in neurological disorders. *Cold Spring Harb. Perspect. Biol*. **4**, a008839 (2012). 10.1101/cshperspect.a008839 PMC3475163 22983160

[54] Ward, D. M. & Cloonan, S. M. Mitochondrial iron in human health and disease. *Annu. Rev. Physiol.* **81**, 453–482 (2019). 10.1146/annurev-physiol-020518-114742 PMC6641538 30485761

[55] Hamdi, A. et al. Erythroid cell mitochondria receive endosomal iron by a “kiss-and-run” mechanism. *Biochim. Biophys. Acta* **1863**, 2859–2867 (2016). 10.1016/j.bbamcr.2016.09.008 27627839

[56] Ziegler PK (2018). Mitophagy in intestinal epithelial cells triggers adaptive immunity during tumorigenesis. Cell.

[57] Bock FJ, Tait SWG (2020). Mitochondria as multifaceted regulators of cell death. Nat. Rev. Mol. Cell Biol..

[58] Gawlowski T (2012). Modulation of dynamin-related protein 1 (DRP1) function by increased O-linked-beta-N-acetylglucosamine modification (O-GlcNAc) in cardiac myocytes. J. Biol. Chem..

[59] Nie H (2020). O-GlcNAcylation of PGK1 coordinates glycolysis and TCA cycle to promote tumor growth. Nat. Commun..

[60] Youle RJ, Narendra DP (2011). Mechanisms of mitophagy. Nat. Rev. Mol. Cell Biol..

[61] Yuzwa SA (2008). A potent mechanism-inspired O-GlcNAcase inhibitor that blocks phosphorylation of tau in vivo. Nat. Chem. Biol..

[62] Caldwell SA (2010). Nutrient sensor O-GlcNAc transferase regulates breast cancer tumorigenesis through targeting of the oncogenic transcription factor FoxM1. Oncogene.

[63] Liu J (2006). Increased hexosamine biosynthesis and protein O-GlcNAc levels associated with myocardial protection against calcium paradox and ischemia. J. Mol. Cell Cardiol..

[64] McClain, D. A. et al. Altered glycan-dependent signaling induces insulin resistance and hyperleptinemia. *Proc. Natl. Acad. Sci. USA* **99**, 10695–10699 (2002). 10.1073/pnas.152346899 PMC125016 12136128

[65] Fan, Y. et al. The effect of anti-inflammatory properties of ferritin light chain on lipopolysaccharide-induced inflammatory response in murine macrophages. *Biochim. Biophys. Acta* **1843**, 2775–2783 (2014). 10.1016/j.bbamcr.2014.06.015 24983770

