# Supplementary material for: Dynamic O-GlcNAcylation coordinates ferritinophagy and mitophagy to activate ferroptosis
Source: Cell Discov. 2022 May 3;8:40. doi: 10.1038/s41421-022-00390-6 (PMC9065108; doi:10.1038/s41421-022-00390-6)
Supplement: Supplementary file 1 — supplementary information [file 41421_2022_390_MOESM1_ESM.pdf]

Fig. S1. (**a-f**) U2OS cells were treated with ML210 (10 μM), iFSP1 (10 μM), or Erastin (10 μM) for different time as indicated. *O*-GlcNAcylation levels were detected by immunoblotting with antibodies against *O*-GlcNAc and β-actin, and the relative intensity (*O*-GlcNAc/β-actin) was quantified. (**g**, **h**) U2OS cells were transfected with control or GPX4 siRNAs for 48 hr and 72 hr, and *O*-GlcNAcylation levels were detected by immunoblotting and the relative intensity (*O*-GlcNAc/β-actin) was quantified. (**i-l**) HUVEC cells and HT1080 cells were treated with RSL3 for different time as indicated and *O*-GlcNAcylation levels were detected by immunoblotting and the relative intensity (*O*-GlcNAc/β-actin) was quantified. (**m**, **n**) U2OS cells were pre-treated with Liproxstatin-1 (200 nM) for 2 hr and then co-treated with RSL3 for different time as indicated. *O*-GlcNAcylation levels were detected by immunoblotting and the relative intensity (*O*-GlcNAc/β-actin) was quantified. *O*-GlcNAcylation levels of cells treated with RSL3 (blue in Fig.1b and purple in Fig.S1n) from Fig1.a, b was used for comparison. (**o**, **p**) U2OS cells were treated with RSL3 for indicated time and the levels of UDP-GlcNAc were detected by UPLC-MS. (**q**) U2OS cells were treated with RSL3, ML210 or iFSP1 for indicated time and the mRNA levels of GFPT1 were assessed by q-RT-PCR. (**r**) U2OS cells were treated with RSL3 for indicated time and the levels of OGT, OGA, and GFPT1 were detected by immunoblotting. (**s**, **t**) U2OS cells were incubated with OSMI-1 or TMG for 12 hr followed by co-treatment of increasing concentrations of ferroptosis inducers Erastin or Sorafenib for 24 hr. Cell viability was indicated by CCK8. (**u**) U2OS cells were transfected with control or OGT siRNAs for 48 hr, and cell lysates were immunoblotted with antibodies against OGT and β-actin. (**v**) U2OS cells were transfected with control or OGT siRNAs for 48 hr, and then pre-treated with Fer-1 for 2 hr before induction with RSL3 for 6 hr. Cells were stained with Trypan blue solution.

Fig. S2. (**a**) U2OS cells were treated with OSMI-1 or TMG for 24 hr, and cells were harvested and lysed with different lysis buffer as indicated before immunoblotting with antibodies against FTH and β-actin. (**b**) U2OS cells were treated with DMSO (Control) or OSMI-1 for 12 hr, and then induced with RSL3 for different time as indicated. Cells were lysed and immunoblotted with antibodies against FTH and β-actin, and the relative optical intensity (FTH/β-actin) was quantified. (**c**) U2OS cells were treated as indicated for 24 hr and cell lysates were subjected to immunoblotting with antibodies against FTH and β-actin, and the relative optical intensity (FTH/β-actin) was quantified. (**d**) U2OS cells were transfected with control or OGT siRNAs for 48 hr, and cell lysates were subjected to immunoblotting with antibodies against FTH, NCOA4 and β-actin. (**e**) U2OS cells were transfected with GFP-FTH and incubated with DMSO or OSMI-1 for 24 hr. Cells were stained with LysoTracker and subjected to confocal microscopy. (**f**, **g**) HeLa and HUVEC cells were treated with OSMI-1 or TMG for 24 hr, stained with LysoTracker and then subjected to immunofluorescence microscopy with antibodies against Ferritin. (**h**) U2OS cells were transfected with control or NCOA4 siRNAs for 48 hr and subjected to immunoblotting with antibodies against NCOA4 and β-actin. Scale bars, 10 μm. $^{**}p < 0.01$, $^{****}p < 0.0001$. Error bars indicate SD.

Fig. S3. (**a**) U2OS cells were stained with FerroOrange alone or co-treated with DFO, and subjected to confocal microscopy. (**b**, **c**) U2OS cells were treated with DMSO (Control) or OSMI-1 for 12 hr, and then induced with RSL3 for 6 hr. Cells were stained with FerroOrange and MitoTracker and subjected to confocal microscopy(**b**). FerroOrange fluorescence intensity of each cell was quantified(**c**). (**d**) U2OS cells were treated with OSMI-1 or TMG for 24 hr, and the mitochondrial ferrous iron levels were assessed by flow cytometry using Mito-FerroGreen. (**e**) U2OS cells were treated with DMSO (Control) or OSMI-1 for 12 hr, and then induced with RSL3 for different time as indicated. The mitochondrial ferrous iron levels were assessed by flow cytometry using Mito-FerroGreen. (**f**) U2OS cells were transfected with control or OGT siRNAs for 48 hr, and the mitochondrial ferrous iron levels were assessed by flow cytometry using Mito-FerroGreen. (**g**) U2OS cells were transfected with control or NCOA4 siRNAs for 48 hr, and treated with DMSO (Control) or OSMI-1 for 24 hr. The mitochondrial ferrous iron levels

were assessed by flow cytometry using Mito-FerroGreen. Scale bars, 10 μm.**p < 0.01, ****p < 0.0001. Error bars indicate SD.

Fig. S4. (**a**, **b**) U2OS cells were treated with OSMI-1 or TMG for 24 hr (**a**), or transfected with OGT or OGA siRNAs for 48 hr (**b**). Cells were subjected to immunofluorescence microscopy with antibodies against TOM20. (**c**, **d**) U2OS cells were treated with OSMI-1 or TMG for the indicated time, and subjected to immunofluorescence microscopy with antibodies against TOM20 and HSP60(**c**). The percentage of unfragmented mitochondria was quantified (**d**). (**e**) U2OS cells were transfected with control or OGT siRNAs for 48 hr and cell lysates were subjected to immunoblotting. (**f-h**) U2OS cells were treated with OSMI-1 or TMG for 24 hr and subjected to immunofluorescence microscopy with antibodies as indicated. (**i**, **j**) HeLa and HUVEC cells were treated with OSMI-1 or TMG for 24 hr and subjected to immunofluorescence microscopy with antibodies against TOM20 and LC3. (**k**) U2OS cells were transfected with control or PINK1 siRNAs for 48 hr and cell lysates were subjected to immunoblotting. Scale bars, 10 μm.

Fig. S5. (**a**) The relative intensity of proteins from Fig 5.A was quantified. (**b**) U2OS cells were transfected with control or combination of PINK1 and NCOA4 siRNAs for 48 hr and then subjected to immunoblotting.

Fig. S6. (**a**) U2OS cells were treated as indicated for 24 hr, stained with LysoTracker and then subjected to immunofluorescence microscopy with antibodies against ferritin and NCOA4. (**b**) 293T cells were transfected with control, OGT or OGA siRNAs for 24 hr and then transfected with indicated plasmids for another 24 hr. Cell lysates were immunoprecipitated with anti-GFP magnetic beads and immunoblotted with the indicated antibodies. (**c**, **d**) 293T cells were transfected with indicated plasmids for 24 hr and the cell lysates were immunoprecipitated with anti-GFP magnetic beads and immunoblotted with the indicated antibodies. (**e**) *In vitro O*-GlcNAcylation assay of FTH was performed and the reaction mixtures were then subjected to co-IP with NCOA4-Myc immunoprecipitated from 293T cells and immunoblotted with the indicated antibodies. (**f**) 293T cells were transfected with indicated plasmids and treated with OSMI-1 or TMG for 24 hr and the cell lysates were immunoprecipitated with anti-GFP magnetic beads and immunoblotted with the indicated antibodies. (**g**) 293T cells were transfected with control, OGT or OGA siRNAs for 24 hr and then transfected with indicated plasmids for another 24 hr and the cell lysates were immunoprecipitated with anti-GFP magnetic beads and immunoblotted with the indicated antibodies. (**h**) 293T cells were transfected with indicated plasmids and treated with OSMI-1 or TMG for 24 hr and the cell lysates were immunoprecipitated with anti-GFP magnetic beads and immunoblotted with the indicated antibodies. (**i**) 293T cells were transfected with control, OGT or OGA siRNAs for 24 hr and then transfected with indicated plasmids for another 24 hr and the cell lysates were immunoprecipitated with anti-GFP magnetic beads and immunoblotted with the indicated antibodies. (**j**, **k**) 293T cells were transfected with indicated plasmids for 24 hr and the cell lysates were immunoprecipitated with anti-Flag or anti-GFP magnetic beads and immunoblotted with the indicated antibodies. (**l**, **m**) Cell lysates from 293T cells overexpressing GFP-FTH-S179A plasmids treated with RSL3 for indicated time were immunoprecipitated with anti-GFP magnetic beads and immunoblotted with the indicated antibodies. The relative optical intensity was quantified. (**n**) U2OS cells were transfected with control or NCOA4 siRNAs for 48 hr and then transfected with GFP-FTH-S179A for 24 hr. Cells were stained with LysoTracker and immunostained with NCOA4 antibody, and then subjected to confocal microscopy.

S1

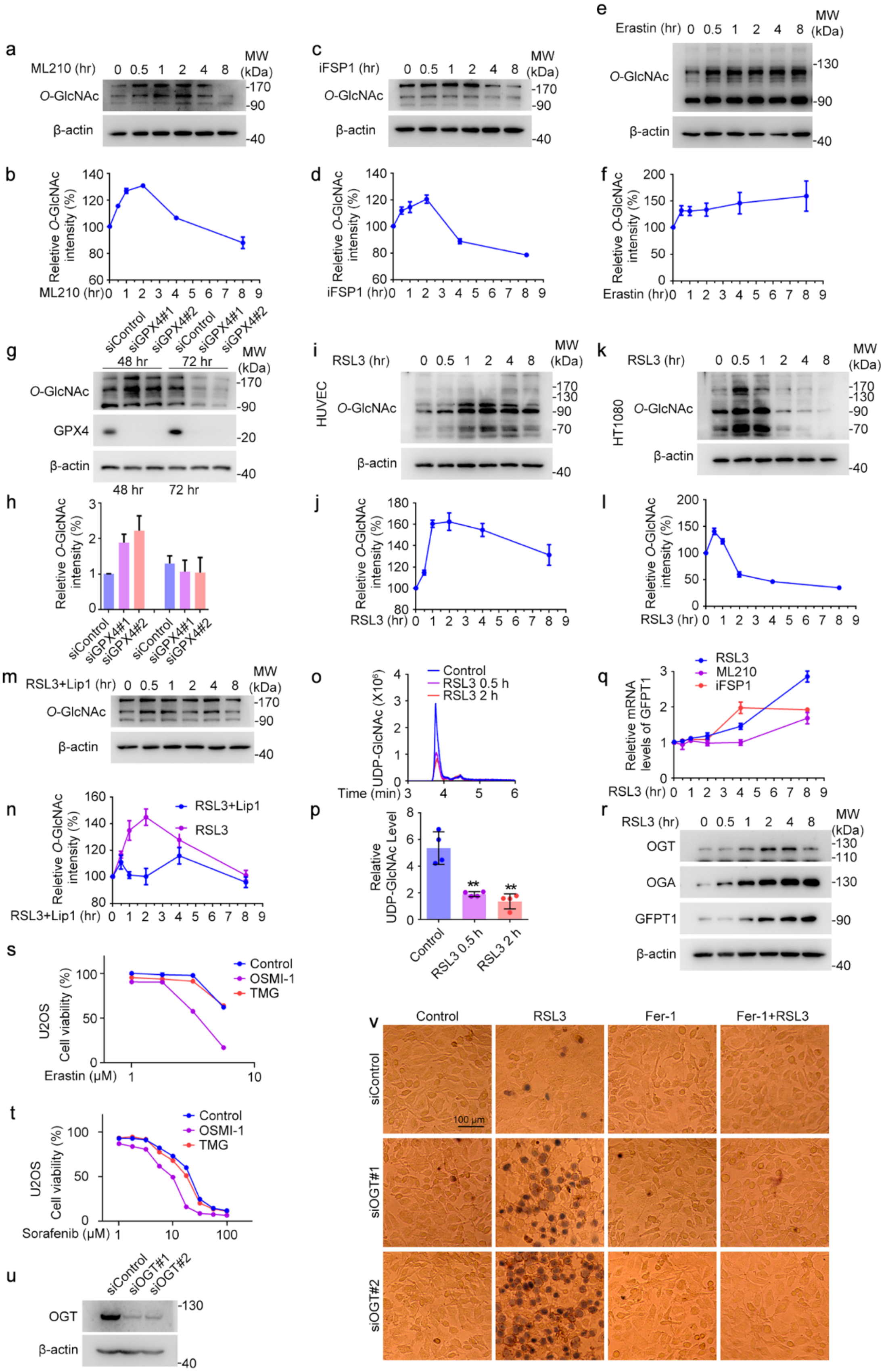

a
ML210 (hr) 0 0.5 1 2 4 8
MW (kDa)
O-GlcNAc
β-actin
b
Reletive O-GlcNAc intensity (%)
ML210 (hr)
c
iFSP1 (hr) 0 0.5 1 2 4 8
d
iFSP1 (hr)
e
Erastin (hr) 0 0.5 1 2 4 8
f
Erastin (hr)
g
siControl
siGPX4#1
siGPX4#2
48 hr
72 hr
GPX4
h
i
RSL3 (hr) 0 0.5 1 2 4 8
HUVEC
j
RSL3 (hr)
k
HT1080
l
m
RSL3+Lip1 (hr) 0 0.5 1 2 4 8
n
RSL3+Lip1
RSL3
RSL3+Lip1 (hr)
o
Control
RSL3 0.5 h
RSL3 2 h
UDP-GlcNAc (X10⁶)
Time (min)
p
Relative UDP-GlcNAc Level
q
RSL3
ML210
iFSP1
Relative mRNA levels of GFPT1
r
OGT
OGA
GFPT1
s
Control
OSMI-1
TMG
U2OS Cell viability (%)
Erastin (μM)
t
Sorafenib (μM)
u
siOGT#1
siOGT#2
v
Control
RSL3
Fer-1
Fer-1+RSL3
100 μm

S2

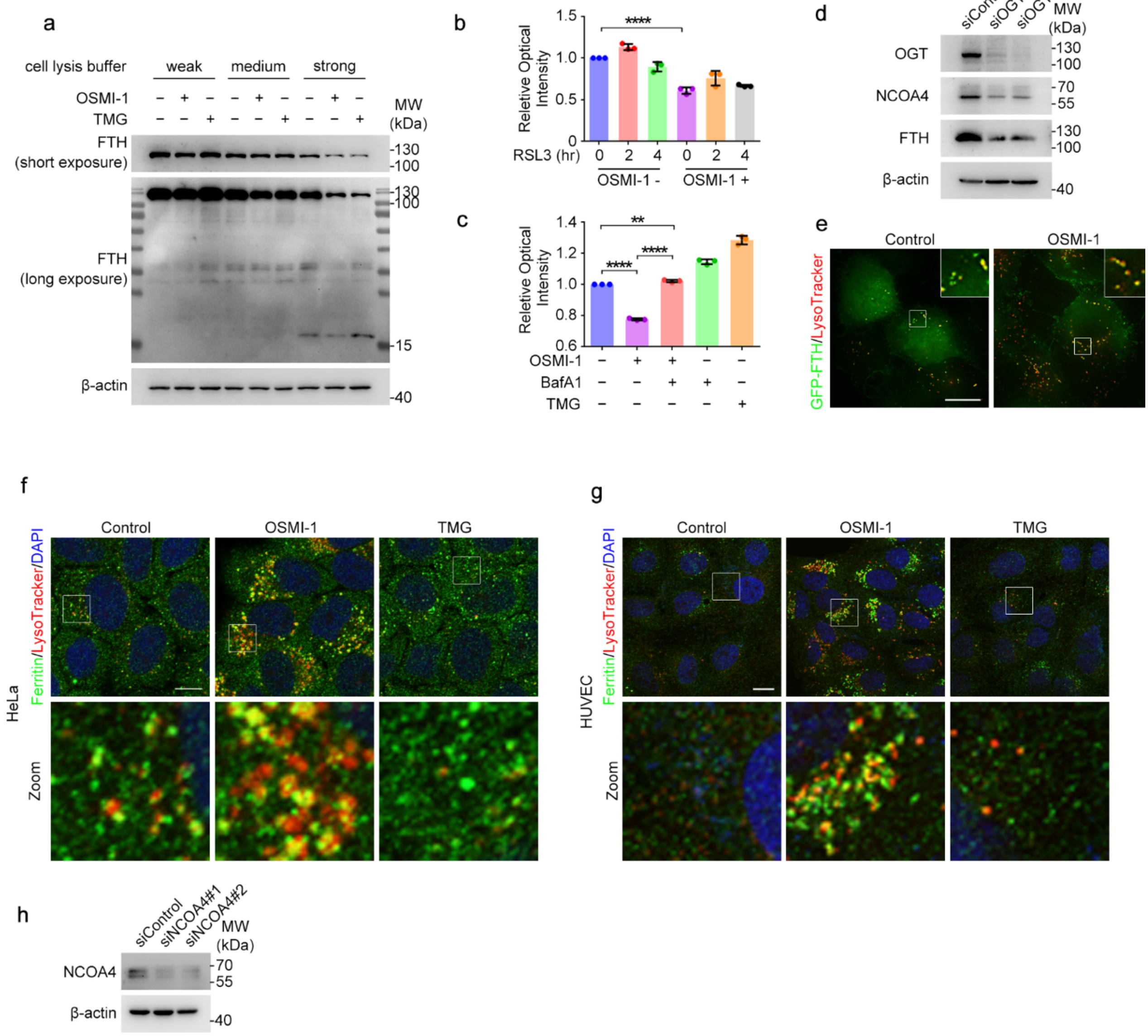

a
cell lysis buffer
weak
medium
strong
OSMI-1
TMG
MW (kDa)
FTH (short exposure)
FTH (long exposure)
β-actin
b
Reletive Optical Intensity
RSL3 (hr)
OSMI-1 -
OSMI-1 +
c
OSMI-1
BafA1
TMG
d
siControl
siOGT#1
siOGT#2
OGT
NCOA4
FTH
β-actin
e
GFP-FTH/LysoTracker
Control
OSMI-1
f
HeLa
Ferritin/LysoTracker/DAPI
Zoom
Control
OSMI-1
TMG
g
HUVEC
h
siNCOA4#1
siNCOA4#2

S3

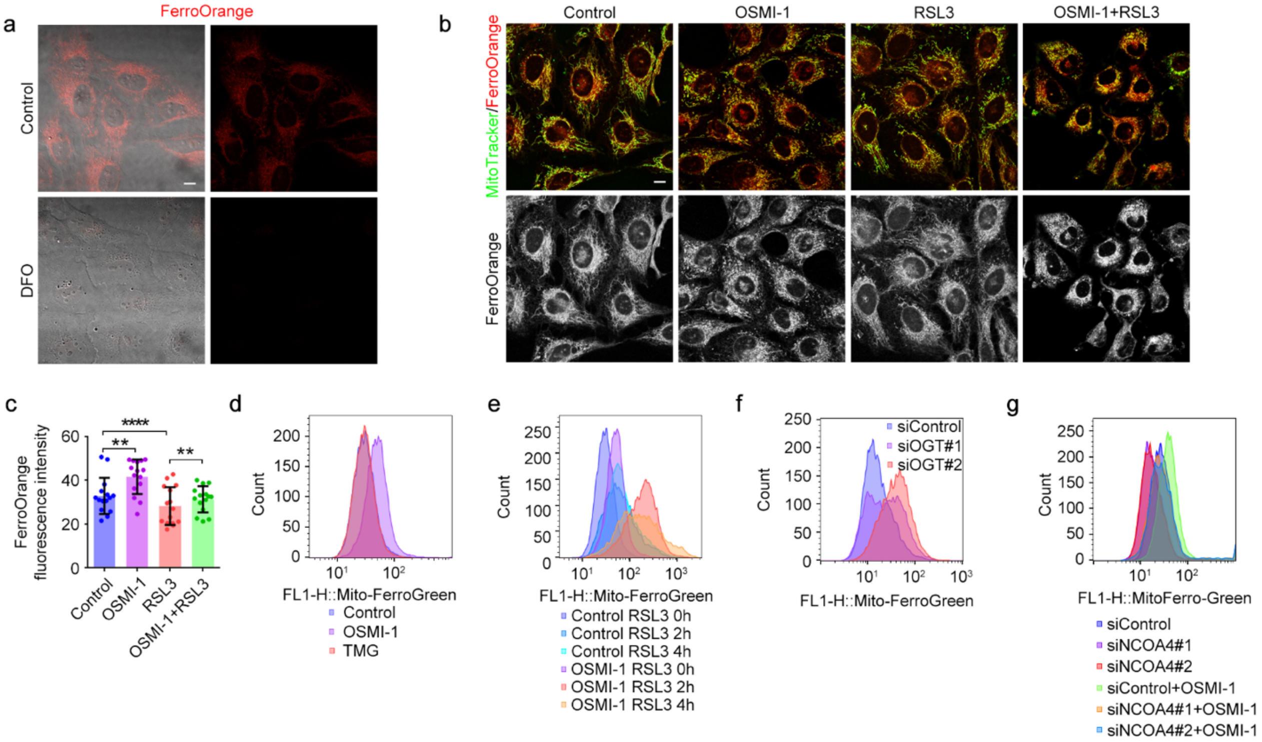
a
FerroOrange
Control
DFO
b
Control
OSMI-1
RSL3
OSMI-1+RSL3
MitoTracker/FerroOrange
FerroOrange
c
FerroOrange fluorescence intensity
****
**
**
Control
OSMI-1
RSL3
OSMI-1+RSL3
d
Count
FL1-H::Mito-FerroGreen
Control
OSMI-1
TMG
e
Count
FL1-H::Mito-FerroGreen
Control RSL3 0h
Control RSL3 2h
Control RSL3 4h
OSMI-1 RSL3 0h
OSMI-1 RSL3 2h
OSMI-1 RSL3 4h
f
Count
siControl
siOGT#1
siOGT#2
FL1-H::Mito-FerroGreen
g
Count
FL1-H::MitoFerro-Green
siControl
siNCOA4#1
siNCOA4#2
siControl+OSMI-1
siNCOA4#1+OSMI-1
siNCOA4#2+OSMI-1

S4

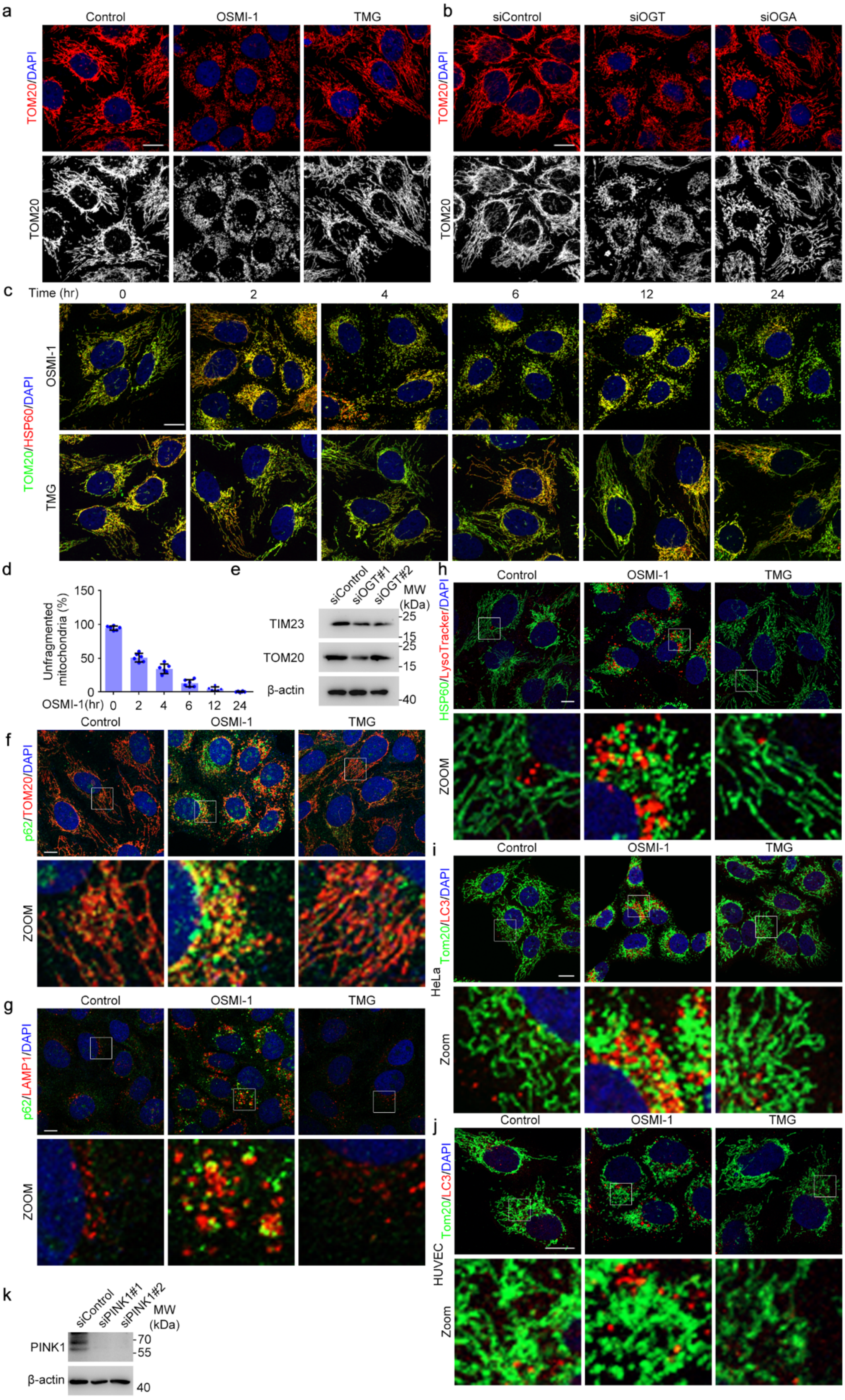
a
Control
OSMI-1
TMG
TOM20/DAPI
TOM20
b
siControl
siOGT
siOGA
TOM20/DAPI
TOM20
c
Time (hr)
0
2
4
6
12
24
OSMI-1
TMG
TOM20/HSP60/DAPI
d
Unfragmented mitochondria (%)
150
100
50
0
OSMI-1(hr) 0 2 4 6 12 24
e
siControl
siOGT#1
siOGT#2
MW (kDa)
TIM23
TOM20
β-actin
-25
-15
-25
-15
-40
h
Control
OSMI-1
TMG
HSP60/LysoTracker/DAPI
ZOOM
f
Control
OSMI-1
TMG
p62/TOM20/DAPI
ZOOM
i
Control
OSMI-1
TMG
HeLa
Tom20/LC3/DAPI
Zoom
g
Control
OSMI-1
TMG
p62/LAMP1/DAPI
ZOOM
j
Control
OSMI-1
TMG
HUVEC
Tom20/LC3/DAPI
Zoom
k
siControl
siPINK1#1
siPINK1#2
MW (kDa)
PINK1
70
55
β-actin
40

S5

A

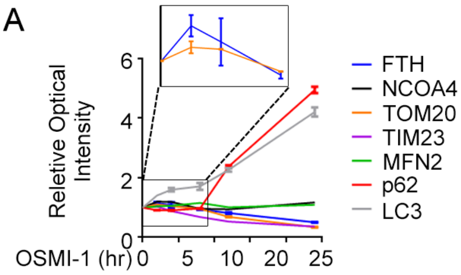

6
4
2
0
Reletive Optical Intensity
OSMI-1 (hr)
0
5
10
20
25
FTH
NCOA4
TOM20
TIM23
MFN2
p62
LC3

B

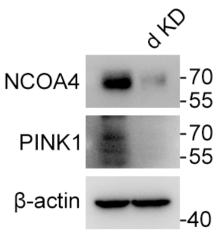

d KD
NCOA4
PINK1
β-actin
70
55
70
55
40

S6

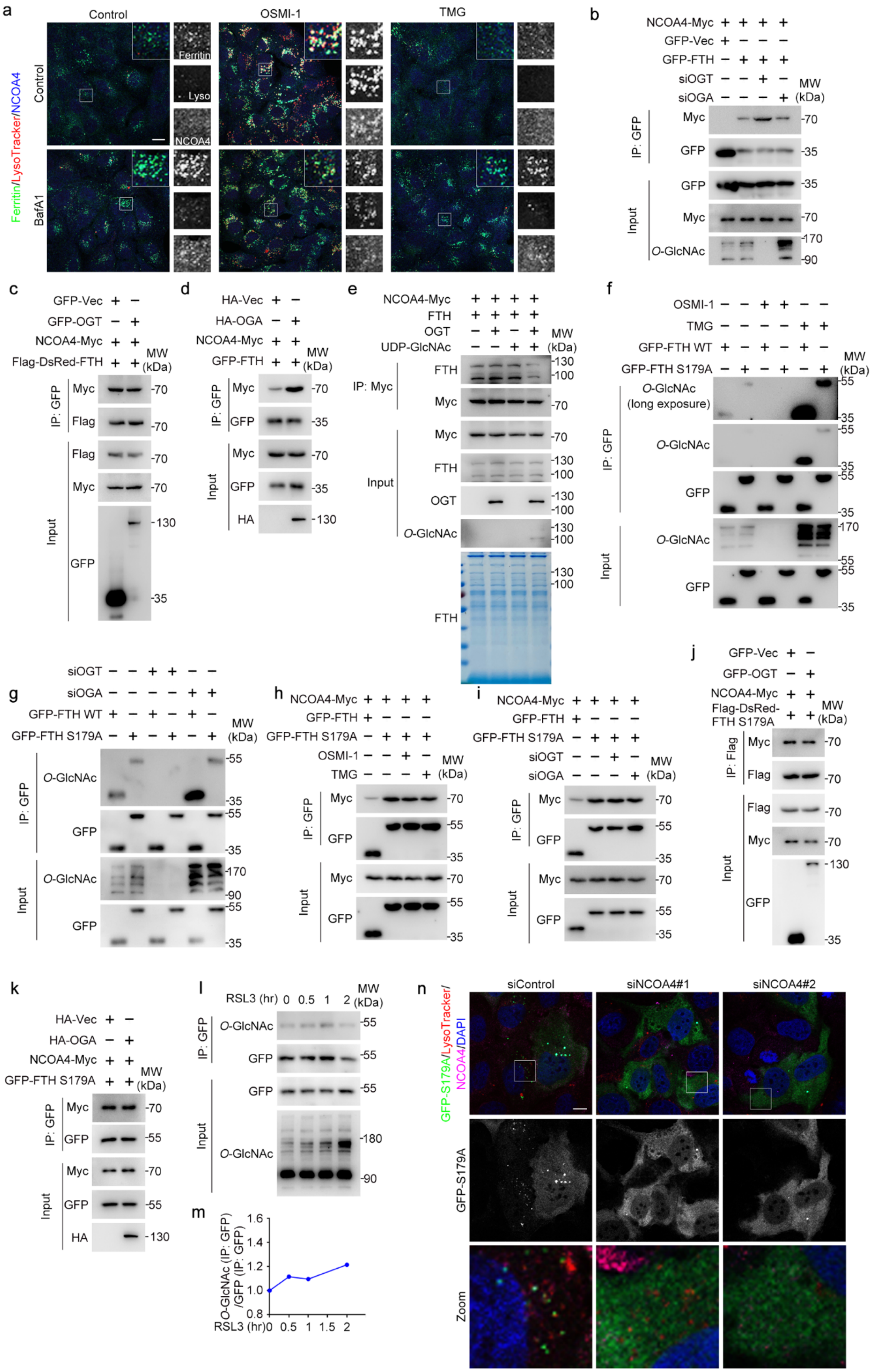

a
Control
OSMI-1
TMG
Ferritin/LysoTracker/NCOA4
Control
BafA1
Ferritin
Lyso
NCOA4
b
NCOA4-Myc
GFP-Vec
GFP-FTH
siOGT
siOGA
MW (kDa)
IP: GFP
Input
Myc
GFP
O-GlcNAc
c
GFP-Vec
GFP-OGT
NCOA4-Myc
Flag-DsRed-FTH
Flag
d
HA-Vec
HA-OGA
GFP-FTH
HA
e
FTH
OGT
UDP-GlcNAc
IP: Myc
f
OSMI-1
TMG
GFP-FTH WT
GFP-FTH S179A
O-GlcNAc (long exposure)
g
h
i
j
Flag-DsRed-FTH S179A
IP: Flag
k
l
RSL3 (hr)
m
O-GlcNAc (IP: GFP) /GFP (IP: GFP)
n
siControl
siNCOA4#1
siNCOA4#2
GFP-S179A/LysoTracker/NCOA4/DAPI
GFP-S179A
Zoom
