# Supplementary material for: Dynamic O-GlcNAcylation coordinates ferritinophagy and mitophagy to activate ferroptosis
Source: Cell Discov. 2022 May 3;8:40. doi: 10.1038/s41421-022-00390-6 (PMC9065108; doi:10.1038/s41421-022-00390-6)
Supplement: Supplementary file 2 — Table S1qPCR primers [file 41421_2022_390_MOESM2_ESM.docx]

Table S1qPCR primers

| β-Actin-F | ATTGGCAATGAGCGGTTCC |
| --- | --- |
| β-Actin-R | GGTAGTTTCGTGGATGCCACA |
| GFPT1-F | AACTACCATGTTCCTCGAACGA |
| GFPT1-R | CTCCATCAAATCCCACACCAG |
| OGT-F | CAGTAGCTTGGAGTAATCTTGGC |
| OGT-R | GGTGACAGCCTTTTCAAAGTGAT |
| OGA-F | CATAGGATGTTTTGGCGAGAGAT |
| OGA-R | GGTGAGATCGCATAGATGAACTC |
| ACSL4-F | CATCCCTGGAGCAGATACTCT |
| ACSL4-R | TCACTTAGGATTTCCCTGGTCC |
| ACSL3-F | GCCGAGTGGATGATAGCTGC |
| ACSL3-R | ATGGCTGGACCTCCTAGAGTG |
| PTGS2-F | CTGGCGCTCAGCCATACAG |
| PTGS2-R | CGCACTTATACTGGTCAAATCCC |
| ALOX15-F | GGGCAAGGAGACAGAACTCAA |
| ALOX15-R | CAGCGGTAACAAGGGAACCT |
| PEBP1-F | CCTGCAAGAAGTGGACGAG |
| PEBP1-R | ACCAAGGTGTAGAGCTTCCCT |
| SLC7A11-F | TCCTGCTTTGGCTCCATGAACG |
| SLC7A11-R | AGAGGAGTGTGCTTGCGGACAT |
| GPX4-F | ACAAGAACGGCTGCGTGGTGAA |
| GPX4-R | GCCACACACTTGTGGAGCTAGA |
| FSP1-F | AGACAGGGTTCGCCAAAAAGA |
| FSP1-R | CAGGTCTATCCCCACTACTAGC |
| CHAC1-F | GAACCCTGGTTACCTGGGC |
| CHAC1-R | CGCAGCAAGTATTCAAGGTTGT |
| SLC39A14-F | AAGGCCCTACTCAACCACCT |
| SLC39A14-R | CGACTGCTCGCTGAAATTGTG |
| TFR-F | ACCATTGTCATATACCCGGTTCA |
| TFR-R | CAATAGCCCAAGTAGCCAATCAT |
| FTH-F | CCCCCATTTGTGTGACTTCAT |
| FTH-R | GCCCGAGGCTTAGCTTTCATT |
| FTL-F | CAGCCTGGTCAATTTGTACCT |
| FTL-R | GCCAATTCGCGGAAGAAGTG |
| FPN1-F | CTACTTGGGGAGATCGGATGT |
| FPN1-R | CTGGGCCACTTTAAGTCTAGC |
| DMT1-F | TGGAGATCATGGGGAGTCTG |
| DMT1-R | AAGAAAACCTGGTCCGGTGAA |
| IRP2-F | TCGATGTATCTAAACTTGGCACC |
| IRP2-R | GCCATCACAATTTCGTACAGCAG |
| NCOA4-F | ACAGTTGCATAAGCCGTCACC |
| NCOA4-R | TGAGCCTGCTGTTGAAGTGTC |
